# 3D Printing Assisted Wearable and Implantable Biosensors

**DOI:** 10.3390/bios15090619

**Published:** 2025-09-17

**Authors:** Somnath Maji, Myounggyu Kwak, Reetesh Kumar, Hyungseok Lee

**Affiliations:** 1 Department of Radiology, University of Michigan, Ann Arbor, MI 48109, USA; smaji@umich.edu; 2 Department of Smart Health Science and Technology, Kangwon National University (KNU), Chuncheon-si 24341, Republic of Korea; eric7947@kangwon.ac.kr; 3 Department of Bioengineering and Biotechnology, School of Basic and Applied Sciences, Galgotias University, Greater Noida 203201, India; reetesh.kumar@galgotiasuniversity.edu.in; 4 Department of Mechanical and Biomedical, Mechatronics Engineering, Kangwon National University (KNU), Chuncheon-si 24341, Republic of Korea

**Keywords:** 3D printing, additive manufacturing, wearable biosensors, implantable biosensors, biocompatible materials, flexible electronics

## Abstract

Biosensors have undergone transformative advancements, evolving into sophisticated wearable and implantable devices capable of real-time health monitoring. Traditional manufacturing methods, however, face limitations in scalability, cost, and design complexity, particularly for miniaturized, multifunctional biosensors. The integration of 3D printing technology addresses these challenges by enabling rapid prototyping, customization, and the production of intricate geometries with high precision. This review explores how additive manufacturing techniques facilitate the fabrication of flexible, stretchable, and biocompatible biosensors. By incorporating advanced materials like conductive polymers, nanocomposites, and hydrogels, 3D-printed biosensors achieve enhanced sensitivity, durability, and seamless integration with biological systems. Innovations such as biodegradable substrates and multi-material printing further expand applications in continuous glucose monitoring, neural interfaces, and point-of-care diagnostics. Despite challenges in material optimization and regulatory standardization, the convergence of 3D printing with nanotechnology and smart diagnostics heralds a new era of personalized, proactive healthcare, offering scalable solutions for both clinical and remote settings. This synthesis underscores the pivotal role of additive manufacturing in advancing wearable and implantable biosensor technology, paving the way for next-generation devices that prioritize patient-specific care and real-time health management.

## 1. Introduction to Biosensors

**Figure 1:**
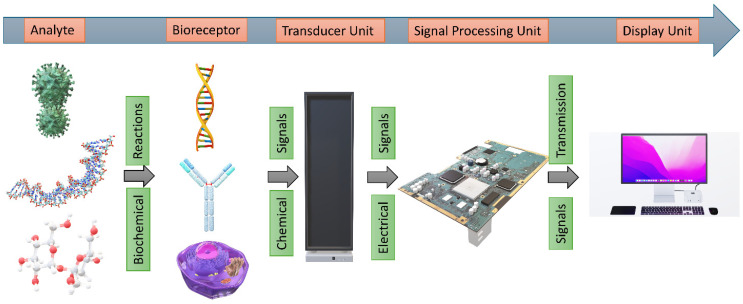
Schematic representation of biosensor operating principles: detection of the target analyte by a specific receptor molecule, followed by signal transduction and output generation.

Biosensors are sophisticated analytical devices that combine biological recognition elements with electronic systems (transducers) to detect and measure specific biological and chemical substances [1]. These devices operate by converting biological responses into electrical signals, enabling the quantitative analysis of various analytes. Their significance spans multiple sectors, including healthcare, environmental monitoring, food safety, and biotechnology, due to their capacity for real-time, sensitive, and accurate detection [2]. Although biosensors contain many components (such as an immobilization matrix, signal processor, and display unit), the main core components that it operates on are a biological recognition element (bioreceptor) and a transducer (Figure 1). Upon introduction of the target molecule (analyte), an interaction occurs with the bioreceptor, leading to biochemical changes that are crucial for signal transduction. These changes are detected by a transducer, which converts the biochemical response into an electrical signal, thus quantifying the concentration of the analyte in question [3]. With abilities to detect low concentrations of analyte, portability, rapid results, and low costs have made them valuable tools in various fields, including healthcare, environmental monitoring, and food safety. Biosensors can be divided into several categories based on their components, detection methods, and target applications (Table 1). Table 1 presents the common target analytes and their detection strategies in modern biosensors. Biosensors can be divided into several categories based on their components, detection methods, and target applications, which are presented in the Supplementary Materials (Table S1). 

**Figure 2:**
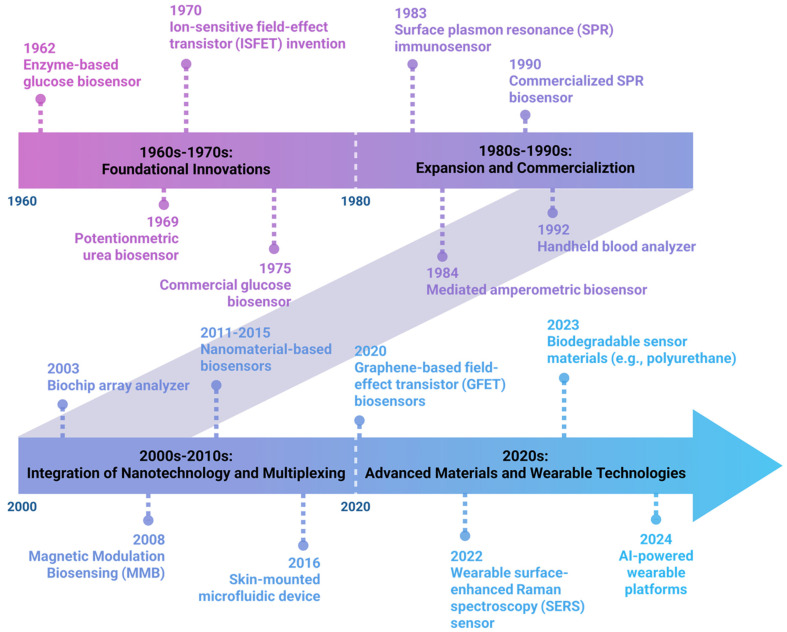
Timeline and key developments in biosensor technology. The field began with Leland C. Clark Jr.’s enzyme-based glucose biosensor (1962) [26], followed by the first potentiometric urea biosensor by Guilbault and Montalvo (1969) [29], and Piet Bergveld’s invention of the ISFET for ion detection (1970) [52]. The first commercial glucose biosensor was launched by Yellow Springs Instruments (1975) [53]. The era of expansion saw the development of the first surface plasmon resonance (SPR) immunosensor (1983) [54] and mediated amperometric biosensors (1984) [27], culminating in the commercialization of SPR-based biosensors (1990) [55] and handheld blood analyzers (1992) [56]. The 21st century marked integration with nanotechnology, multiplexing, and wearable formats, such as the biochip array analyzer (2003) [57], magnetic modulation biosensing for enhanced sensitivity (2008) [58], and nanomaterial-based sensors (2011–2015) [59]. The introduction of skin-mounted microfluidic devices (2016) [60], ultra-sensitive graphene-based FET biosensors (2020) [61], and wearable SERS sensors (2022) [62] facilitated non-invasive diagnostics. Recent innovations include biodegradable sensor materials (2023) [63] and AI-powered wearable platforms for biomedical applications (2024) [64].

The story of biosensors begins in 1956 with Leland C. Clark, often called the “Father of Biosensors” who laid the groundwork for modern biosensors (Figure 2). Clark’s breakthrough came in 1962 when he modified his oxygen electrode to detect glucose. He did this by trapping glucose oxidase (an enzyme) against the electrode using a dialysis membrane. This created the first true biosensor, which worked by measuring how much oxygen was consumed when the enzyme reacted with glucose [25,26]. This fundamental design—combining a biological component with an electrode—established the basic principle that biosensors still use today. Later, it was observed that the first-generation biosensors faced critical oxygen dependency issues, where glucose oxidase relied on dissolved oxygen as the natural electron acceptor, making measurements highly susceptible to fluctuating oxygen concentrations in biological environments. This limitation rendered these sensors unreliable for in vivo applications, particularly in oxygen-deficient tissues or during physiological changes affecting oxygen availability. In the next couple of decades (1970s–1980s; often termed as the second-generation biosensors), scientists made biosensors more efficient by improving how signals were detected. Instead of measuring oxygen consumption, they developed ways to directly detect the electrons produced in biological reactions. For example, derivatives of ferrocene (an introduction of artificial electron mediators) replaced oxygen as electron acceptors, thereby improving electron transfer efficiency, reducing dependence on environmental oxygen, and enabling more reliable amperometric detection [27,28]. Some other developments of new immobilization techniques [29,30], such as the integration of enzymes with microelectrodes, facilitated the miniaturization and creation of the first commercial biosensors (developed by Yellow Spring Instruments, YSI) primarily for glucose monitoring in diabetes management. While this innovation significantly improved reproducibility and enabled the development of commercial glucose test strips, second-generation systems introduced new limitations, including mediator leaching, potential toxicity, and restricted applicability for implantable devices. The next generation brought about “reagent less” biosensors, where the biological component was directly connected to the electrode [31,32]. Some other novel introductions, like conducting polymers [33,34], new biological recognition elements [35], and integration with microelectronics and miniaturization techniques occurred [36]. The post-2000s era, often referred to as the “modern era” of biosensing, marks the convergence of multiple enabling technologies. A central driver has been the incorporation of nanomaterials, such as quantum dots, nanoparticles, graphene, and carbon nanotubes. The introduction of nanomaterials dramatically increased the surface area-to-volume ratio, exponentially enhancing the available active sites for biomolecule immobilization and catalytic reactions [37]. This significantly enhanced sensor sensitivity and enabled the detection of extremely small quantities of target molecules [38,39]. Nanomaterials facilitate a direct electron transfer (DET) between enzymes and electrodes, eliminating the need for mediators entirely in previous-generation biosensors. This was achieved through the unique electronic properties of nanomaterials, particularly their enhanced electrical conductivity and favorable electron transfer kinetics [40]. The quantum confinement effects in nanomaterials such as quantum dots introduced tunable optical and electronic properties that were impossible to achieve with bulk materials [41]. These advances solved the core miniaturization challenge that had previously confined diagnostic testing to centralized laboratories and allowed healthcare providers to conduct real-time diagnostics at the patient’s bedside, in remote areas, or in resource-limited settings [42,43]. Consequently, this shift has catalyzed the development of smart biosensors that are capable of continuous, multiplexed monitoring, wireless data transmission, and seamless integration into healthcare systems [44,45].

As demand grew for continuous and non-invasive monitoring, biocompatible flexible substrates and adhesives such as silicone, hydrogel, and natural biomaterials (e.g., silk fibroin, cellulose) were engineered to conform to the skin, mitigate irritation, and maintain sensor performance even with movement [46]. These advances were essential for transitioning biosensors from intermittent use to flexible patches, textiles, and accessories integrated into everyday life [47]. Innovations in bioinspired and biomineralized coatings, as well as biodegradable polymers, allowed the development of implantable sensors to remain functional inside the body for months while safely interfacing with tissues [48]. Engineered bioinks and 3D-printed biocompatible matrices further improved integration and the miniaturization needed for chronic implants. This material progress transformed biosensors from sporadic standalone tools to seamless health platforms embedded in daily life and clinical practice [49,50,51].

## 2. Wearable and Implantable Biosensors

Wearable biosensors are portable devices that are designed to be worn on, inside, or near the human body to continuously monitor physiological or biochemical parameters that indicate a patient’s health status. These sensors provide real-time measurements of physiological parameters, generating digital outputs that are easy to interpret and act upon. By allowing patients quick access to clinical information, these devices promote proactive health management in a more convenient and cost-effective manner, improving user compliance [65,66]. In recent years, various wearable technologies have been introduced in scientific research, particularly for personalized medicine, point-of-care diagnostics, and fitness or home health monitoring. These platforms collect physiological data through wearable components such as patches [67], lenses [68], headbands [69], wristbands [70], eyeglasses [71], and skin-conformal tattoos [72]. They are capable of detecting a range of health indicators, including glucose levels, blood pressure, heart rate, oxygen saturation, respiratory and tactile parameters, body motion, skin temperature, and brain activity [73].

Although wearable biosensing devices have become popular with their real-time and quick measurements that are easy to interpret and act upon, there are certain challenges with wearable biosensing devices, such as they are unfeasible for providing data by penetrating deep into the tissue, while contaminants from the environment might affect the results, and only global information is provided. Therefore, in addition to these flexible wearable biosensing devices, there is also an urgent need to have easy implantable devices that can bring forth the efficacious pathway of not only better diagnostic but also therapeutic options. For example, cardiac monitors have revolutionized cardiology by providing continuous heart rhythm tracking, allowing for early detection and intervention in life-threatening arrhythmias [74]. Implantable pressure sensors have facilitated the management of conditions such as hydrocephalus and traumatic brain injury by allowing for continuous intracranial pressure monitoring [75]. These advancements highlight the adaptability and transformative impact of implantable sensors in tackling various health conditions, paving the way for a new era of personalized and proactive healthcare.

The growing acceptance of wearable and implantable biosensors is driven by advancements in fabrication techniques that enable features like flexibility, stretchability, ultra-thinness, and lightweight designs, ensuring a seamless integration with the body [76,77]. Considerable research has focused on enhancing the interaction between these multifunctional devices and biological systems. Inspired by the properties of soft materials, ultra-thin biosensors are designed to conform to biological surfaces for improved performance [78,79,80]. Currently, a variety of fabrication processes, including lithography-based techniques, microchannel molding, and deposition techniques such as vapor or electrochemical deposition, laser ablation, roll-to-roll printing, and micromachining, are used to build the sensing interfaces [81,82,83]. However, there are many challenges that this technique faces in fabricating sensitive and flexible biological sensors, such as limited manufacturing scalability, high production costs, reduced durability, and restricted adaptability, which continue to hinder the widespread adoption of these cutting-edge wearable biosensors [84,85]. The shortcomings of conventional manufacturing methods have been addressed by additive manufacturing (AM), sometimes referred to as 3D printing, which has grown substantially in recent years.

## 3. The Requirement of 3D Printing Technology

Three-dimensional printing, a form of additive manufacturing, has revolutionized every manufacturing sector. Its ability to produce completely three-dimensional structures with intricate features in a single step makes it particularly appealing [86]. Charles Hull was the first to create this technique in 1986 [87]. Three-dimensional printing uses computer-controlled procedures based on three-dimensional (3D) digital representations of the item to be printed to fabricate a variety of structures by printing successive layers of materials that are built on top of one another. The academic and corporate communities have recently shown a great deal of interest in additive manufacturing, and it has been referred to as a third industrial revolution [86,88]. According to market research, the worldwide 3D printing market was valued at USD 16.54 billion in 2021 and is projected to increase at a CAGR of 21.0% between 2021 and 2028. By 2028, the worldwide market is expected to grow to over USD 63 billion [89]. Biomedical applications [90,91], electronics and sensors [92], lightweight engineering materials [93,94,95], and multifunctional composites [96,97,98] are just a few of the many fields in which it has attracted industry and scientific attention.

**Figure 3:**
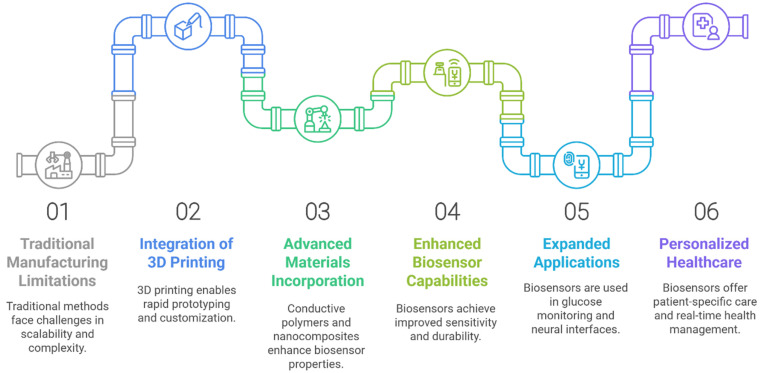
Evolution of biosensors with the integration of 3D printing technologies. The schematic illustrates the progression from traditional manufacturing limitations to the era of personalized healthcare. Key milestones include the adoption of 3D printing for rapid prototyping, incorporation of advanced functional materials, enhanced sensor performance, and expansion into diverse biomedical applications such as glucose monitoring and neural interfaces—ultimately enabling patient-specific, real-time health management. This trajectory highlights the transformative impact of 3D printing in developing next-generation wearable and implantable biosensors.

In the production of biosensors or biosensor components, 3D printing technologies present a promising advance. As mentioned above, wearable biosensors have multiple miniaturized complex structures, which are difficult to fabricate in a single unit using conventional manufacturing techniques like coating and injection molding. Three-dimensional printing offers numerous benefits over traditional manufacturing, such as increased versatility, reduced waste, greater design freedom, low fabrication costs, high automation, and a short fabrication cycle time [87,99]. In this regard, several unique aspects of 3D printing have found their way into the fabrication of biomedical sensors. For instance, 3D printing streamlines the production process by using material deposition and curing/sintering to create the individual components. Secondly, the miniaturization and compactness of the biosensors were easily achievable with the use of 3D printing, as it enables the integration of different materials during the fabrication process. For example, by integrating many sensing modalities into a single sensor via extrusion printing, researchers have achieved downsizing by lowering the device’s total footprint in comparison to using separate sensors [100]. Also, through the progressive addition of material digitally controlled by computer-aided design (CAD) systems, 3D printing enables the very precise fabrication of personalized parts [101]. Another example showcasing the 3D printing capability of tackling the problem of complicated geometries is the development of Clark platinum electrodes. Previously, the Clark platinum electrodes with special shapes were developed for ongoing oxygen concentration measurement in cardiovascular surgery. However, because each sensory component was made individually and manually integrated, these sensors were challenging to mass-produce. Later, 3D printing was used to tackle the problem of developing the multi-component complicated shapes of the electrodes as a single unit. Product customization poses a difficulty for conventional manufacturers, mostly due to the substantial expenses associated with mold fabrication, particularly for small-scale manufacturing of bespoke items. Conversely, 3D printing can produce limited numbers of tailored plastic objects at far lower prices than conventional mold-based manufacturing. This is particularly beneficial in biomedical disciplines, where individualized patient-specific goods are also necessary [87]. By combining complex geometries into specific microstructures, 3D printing enables the on-demand production of customizable sensing devices. Along with miniaturization and customization, the widespread acceptance of 3D printing relies on the fabrication of materials with critical characteristics such as stretchability, flexibility, ultra-thinness, and lightweight that fuel the development of more efficient wearable and implantable platforms (Figure 3) [73,102]. In recent years, 3D printing has become more popular in academic research because of its ease in rapid manufacturing when dealing with emerging multifunctional materials [103,104]. Interestingly, these sophisticated multifunctional and multipurpose materials are being investigated for use in 3D-printed bio-integrated devices to give doctors, patients, healthcare professionals, and healthy individuals ways to track their health. Through the use of diverse soft functional materials, 3D printing technology may enable the meticulous creation of patient-specific geometry in the context of wearable biomedical devices, directly on the preferred surfaces [85,97,103,105].

This review highlights the latest developments in 3D-printed bio-integrated sensor technologies, with a focus on wearable and implantable biosensors. We begin by exploring how innovative 3D printing techniques are enabling the fabrication of flexible 3D structures using advanced printable soft materials. Subsequently, we emphasize various 3D printing approaches used in the development of wearable and implantable biosensors. Later, this article concentrates on 3D-printed wearable (bio)sensors designed for various applications, such as detecting electrophysiological signals, biochemical signals, and signals from the dynamics of vascular flow patterns. Additionally, readers are directed to several recently published studies on 3D-printed biosensors and (bio)analytical sensors for further exploration.

## 4. 3D Printing—Materials and Methods

**Figure 4:**
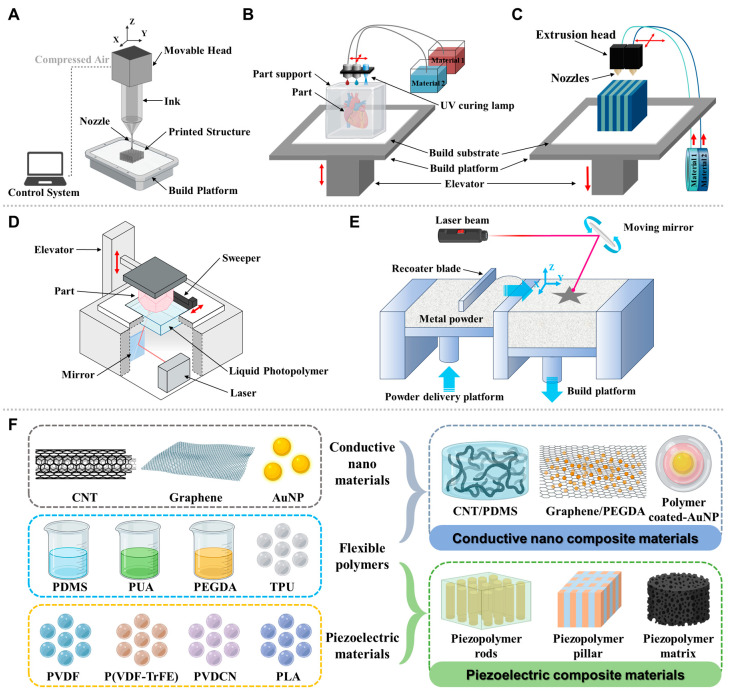
Overview of various 3D printing techniques and printable materials for the fabrication of wearable biosensors: (**A**) Direct ink writing (DIW); (**B**) inkjet printing; (**C**) fused filament fabrication (FFF); (**D**) stereolithography (SLA); (**E**) selective laser melting (SLM). Printable functional materials include (**F**) conductive nanocomposite materials and piezoelectric composite materials, which enable the development of flexible and high-performance wearable bioelectronics. Abbreviations: Carbon nanotube—CNT; gold nanoparticle—AuNP; polydimethylsiloxane—PDMS; polyurethane acrylate—PUA; polyethylene glycol diacrylate—PEGDA; Thermoplastic polyurethane—TPU; polyvinylidene fluoride—PVDF; poly (vinylidene fluoride-trifluoroethylene)—P (VDF-TrFE); polyvinylidene chloride—PVDCN; poly lactic acid—PLA.

Regarding key 3D printing technologies for wearable biosensor fabrication, several 3D printing technologies have emerged as particularly suitable for wearable biosensor fabrication, each offering distinct advantages for specific sensing applications (Figure 4). Direct ink writing (DIW) has emerged as a versatile 3D printing technique for wearable biosensor fabrication, offering exceptional control over material deposition and structural features. This approach employs computer-controlled extrusion of functional inks through fine nozzles to create precise patterns of sensing elements and supporting structures [106]. This technology enables the incorporation of nanomaterials, including carbon nanotubes, graphene, and metallic nanoparticles, into inks with carefully tailored rheological properties, resulting in high-performance electrodes with enhanced conductivity and sensing capabilities [106,107]. One significant advantage of DIW is its compatibility with a wide range of substrate materials, including flexible polymers, hydrogels that conform to body contours, facilitating improved sensor-skin interfaces and enhanced signal acquisition [108]. DIW also enables the incorporation of nanomaterials, including carbon nanotubes, graphene, and metallic nanoparticles, into inks with carefully tailored rheological properties, resulting in high-performance electrodes with enhanced conductivity and sensing capabilities [106,107]. Unlike high-temperature FDM or laser-based SLS, DIW can work with low-temperature printing that allows compatibility with biomolecules and living cells. Another wide approach that inkjet printing technology offers is the unique advantages for wearable biosensor fabrication, particularly for creating high-resolution conductive traces and precise deposition of functional materials. Inkjet printing eliminates the need for masks, molds, and complex lithography steps, reducing production costs and enabling mass fabrication [109]. It is ideal for rapid prototyping and large-scale manufacturing of biosensors. With droplet volumes in picoliters and feature sizes down to 50–100 µm, inkjet printing achieves fine patterns essential for miniaturized sensors and high-density arrays [106,110]. The non-contact drop-on-demand approach reduces material waste and prevents substrate contamination, crucial for sensitive biological components. It supports sequential printing of diverse materials, including conductors (metal and carbon-based nanoparticles) and biologics (enzymes and small molecules) on flexible substrates (e.g., PDMS, plastics, paper, and textiles) [111,112]. Using high-resolution 3D-scanned body-shape data, researchers have demonstrated the fabrication of on-demand personalized wearable sensors that accurately conform to individual anatomical features [110]. Inkjet-printed electrodes on flexible substrates (e.g., textiles) enable the real-time monitoring of biomarkers in sweat (e.g., lactate and glucose) or electrophysiological signals (ECG and EEG) [113,114]. Extrusion-based approaches, including fused filament fabrication (FFF), provide cost-effective solutions for creating structural components and housings for wearable sensing platforms [115,116]. These methods allow for the integration of different functional materials within a single printing process, enabling the creation of multifunctional sensing systems with improved mechanical and electrical properties. FFF supports a broad range of thermoplastics and composites, including conductive (e.g., Proto-Pasta^®^ CB-PLA, and Black Magic^®^ graphene-PLA), biodegradable (PLA), and biocompatible materials (polycaprolactone, PCL) [117,118]. For example, in one of the studies, FFF was used to print bespoke filaments with optimized conductive filler ratios (PLA (60 wt%) graphite (40 wt%)) to improve electron transfer, enabling detection of biomarkers like the SARS-CoV-2 spike protein at pM levels [119]. A quantitative comparison of various 3D-printed methods used for the fabrication of wearable and implantable biosensors is presented in Table 2.

Materials advances that enable 3D-printed biosensors are a critical component of many 3D-printed biosensors is the electrode, which requires electrically conductive materials. The most common base polymers for these composites include polylactic acid (PLA) and acrylonitrile butadiene styrene (ABS), favored for their compatibility with fused deposition modeling (FDM), a widely accessible 3D printing method [120]. To achieve conductivity, these polymers are embedded with carbon-based materials such as graphene, carbon nanotubes (CNTs), and carbon black [107]. Graphene-layered substrates have been successfully synthesized and integrated into flexible wearable biosensor platforms, providing exceptional electrical conductivity while maintaining the mechanical compliance necessary for skin-interfaced applications [121]. The primary advantage of these composites lies in their ability to create bespoke electrode geometries, enhancing the sensitivity and performance of the resulting biosensor. However, challenges remain in achieving uniform dispersion of conductive fillers within the polymer matrix, which can affect the consistency and reliability of the sensor’s performance. Piezoelectric composites have received widespread attention for their ability to convert mechanical forces into charge signals, making them particularly valuable for motion-sensing applications and energy harvesting in wearable devices [122]. These materials enable self-powered sensing capabilities that reduce or eliminate the need for external power sources.

For biosensors intended for medical applications, particularly those that come into contact with biological fluids or tissues, biocompatibility is paramount. Materials like polycaprolactone (PCL) and various photocurable resins used in stereolithography (SLA) and digital light processing (DLP) are often chosen for their non-toxic and biocompatible properties [123]. In this domain, hydrogels are emerging as a particularly promising class of materials. These water-rich polymer networks are inherently biocompatible and can be 3D-printed with high precision [124]. An example of a hydrogel used for 3D printing biosensors is the conductive GelMA (Gelatin Methacryloyl) hydrogel that is designed for electrochemical sensing. This material combines the excellent biocompatibility of a natural polymer with the electrical properties (by dispersing graphene nanoplatelets) needed for a functional sensor [125]. The field is continuously evolving with the introduction of novel functional polymers. For example, advancements in multi-material 3D printing are enabling the integration of different polymers with distinct properties into a single biosensor, such as combining rigid housing with flexible, conductive sensing elements. One such example is PEDOT:PSS, (poly (3,4-ethylenedioxythiophene):poly (styrene sulfonate)), which stands out as a particularly promising material for wearable health monitors due to its unique combination of excellent conductivity, biocompatibility, and flexibility, which are well-suited for the fabrication of complex, customized bioelectronic devices. PEDOT:PSS’s unique combination of aqueous processability enables its formulation into inks compatible with emerging fabrication technologies like inkjet printing, direct ink writing (DIW), and electrohydrodynamic printing, facilitating cost-effective manufacturing of high-resolution devices. Most significantly, its electrical conductivity is highly tunable through post-treatment with solvents, enabling performance optimization for specific sensing applications, ranging from electrophysiological monitoring to electrochemical biomarker detection [126,127,128]. Recent advancements have focused on enhancing its mechanical durability and stretchability through the formation of composites with elastomers or hydrogels, ensuring robust operation under physical deformation [129,130,131]. Flexible substrate materials such as polydimethylsiloxane (PDMS) serve as ideal platforms for accommodating the complex geometries and integrated functionalities of modern wearable biosensors [132]. These substrates provide the necessary mechanical compliance for maintaining stable contact with the skin during movement while supporting the integration of various sensing modalities and electronic components [100].

Although 3D-printed biosensors have advanced quickly, there are still challenges to overcome. One major issue is the limited availability of high-performance materials made specifically for 3D printing in biosensing. Another important challenge is ensuring that these devices remain stable and reliable over time, especially in complex biological environments. As new polymers and composites with enhanced conductivity, biocompatibility, and functionality are developed, the creation of low-cost, customizable, and highly sensitive wearable and implantable biosensors will become increasingly accessible, paving the way for next-generation healthcare and personalized medicine.

**Table 2:** Quantitative comparison of 3D printing methods over conventional methods in biosensor fabrication.

Fabrication Method	Typical Feature Size/Resolution	Sensor Transduction Type	Sensitivity/Limit of Detection	Fabrication Speed (per Device or Batch)	Approx. Cost per Device (Prototype vs. Scaled)	Notable Advantages/Limitations	References
**SLA/DLP 3D** **Printing**	Sub-50 μm features achievable in some resins; practical ~50–200 μm walls and microchannels	Electrochemical, optical, or impinging microfluidic integration	Limit of quantity in pM–nM range for some electrochemical sensors; depends on electrode surface area and functionalization	Minutes to hours per device for single parts; rapid prototyping; multi-part assemblies possible	Prototype cost is low to moderate; scalable with batch printing	High-resolution smooth surfaces; post-processing (washing, curing, and sealing) can influence performance	[133,134]
**FDM (thermoplastic)**	Typical feature ~100–300 μm; printers ~50–100 μm with high-end nozzles;	Electrochemical, colorimetric, or integrated microfluidics	Limit of quantity often higher than SLA/DLP, but acceptable for glucose, urea, with surface modifications	Slow per device due to layer-by-layer deposition; batch printing feasible for simple housings	Low material cost; high-volume tooling not required; unit cost higher at small runs	Best for rugged housings and disposable cartridges; limited microchannel resolution	[118]
**Powder-** **Based** **Sintering/SLS**	~100–200 μm features; complex geometries possible	Electrochemical, adhered membranes, microfluidic networks	Variable; often in μM–nM for optimized electrode surfaces; not all SLS surfaces are chemically active	Moderate; build time scales with part volume; post-processing (debinding, sintering) adds time	Moderate tooling; no molds, but material costs are higher; post-processing adds steps	Good for robust, solvent-resistant parts; surface chemistry can be challenging	[135]
**Inkjet 3D** **Printing (droplet-based)**	High resolution for membranes and films; ~tens of micrometers in thickness	Optical, colorimetric, enzyme films	Often high sensitivity with surface coatings; limit of detection in μM–nM depending on biofunctionalization	Moderate; drop-on-demand patterns; faster for small arrays	Moderate for consumables; no tooling, scalable for arrays	Flexible sensor patterning and rapid multiplexing	[136]
**Photolithography/Microfabrication**	Sub-micron to micron-scale features (e.g., microfluidic channels)	Electrochemical, optical, and enzymatic	Limit of detection depending on electrode design; e.g., pM–nM range in optimized electrodes	High-volume throughput; batch processing possible	High upfront tooling (photomasks, molds) but very low per-unit cost at scale	Excellent control, repeatability, and scalability; long-established ecosystems	[137]
**Screen** **Printing**	50–200 μm typical channel and electrode features	Electrochemical	Competitive Limit of detection for well-established assays (e.g., glucose) with functionalized inks	High-throughput; rapid batch production	Very low per-unit cost at scale; expensive for molds/tools upfront	Simple, cost-effective for disposable sensors; limited complex 3D geometry	[138]
**Injection** **Molding**	Microfluidic channels down to ~100 μm in optimized molds	Electrochemical, optical	High signal-to-noise with well-defined net surfaces	Very high when production volumes are large	High tooling cost; low per-unit cost at scale	Best for mass production of disposable biosensors; long lead time to set up	[139]

## 5. Applications for Wearable Biosensors by 3D Printing Technology

**Figure 5:**
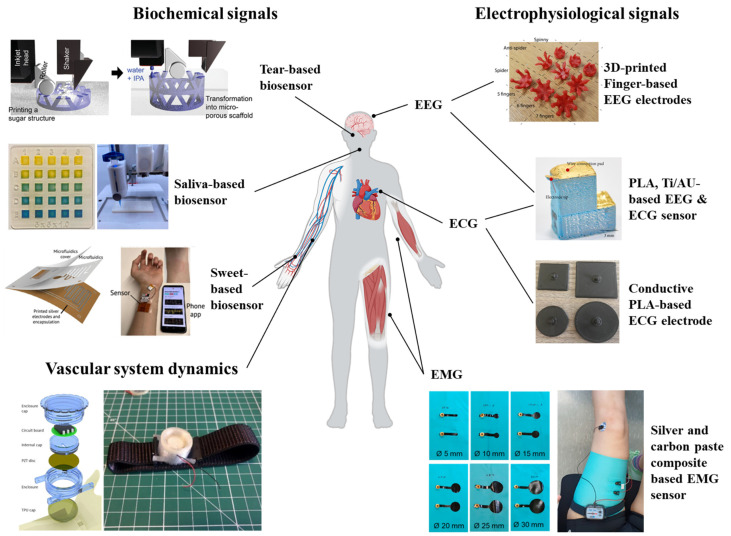
Applications for wearable biosensors by 3D printing technology. Clockwise from top: flexible finger-based EEG electrodes fabricated by FDM printing using TPU (reproduced with permission from [140], copyright 2019, MDPI). EEG and ECG sensors based on PLA and Ti/Au, fabricated using the FDM method (reproduced with permission from [141], copyright 2017, Wiley). Conductive PLA-based ECG electrode fabricated using the FDM method (reproduced with permission from [142], copyright 2023, Wiley). EMG sensors fabricated using a silver and carbon paste composite (reproduced with permission from [143], copyright 2020, MDPI). Wearable biosensor fabricated by FFF printing using piezoelectric materials for continuous monitoring of mechanical vibrations from the artery (reproduced with permission from [144], copyright 2024, MDPI). Wearable sweat sensor with integrated microfluidic channels fabricated by DIW 3D printing (reproduced with permission from [145], copyright 2025, Wiley.). A hydrogel-based colorimetric biosensor fabricated by 3D printing for saliva analysis (reproduced with permission from [146], copyright 2024, MDPI). A conductive sugar scaffold fabrication by inkjet printing for wearable biosensor applications (reproduced with permission from [110], copyright 2020, Wiley).

Three-dimensional-printed biosensors have demonstrated remarkable potential for continuous health monitoring and disease management across various clinical contexts. This continuous real-time monitoring is mostly suitable when used for skin-wearable sensor applications. In this section, we have highlighted some significant contributions of 3D printing technologies towards developing wearable biosensors based on biophysical and biochemical signals. In this review, physiological signals are divided into three subtypes: (i) electrophysiological signals, (ii) biochemical signals, and (iii) vascular systems. A schematic with the application of 3D-printed biosensors on the types of physiological signals is presented in Figure 5.

### 5.1. Electrophysiological Signals

Electrophysiological signals are electrical manifestations of biological processes, particularly from the brain, heart, muscles, and nerves. These signals are crucial for diagnosing and monitoring medical conditions. Depending on the sensing position, it is possible to monitor electrophysiological signals from the skin. Electrocardiography (ECG), electromyography (EMG), and electroencephalography (EEG) are examples of electrophysiological biosignals that are frequently monitored.

**Figure 6:**
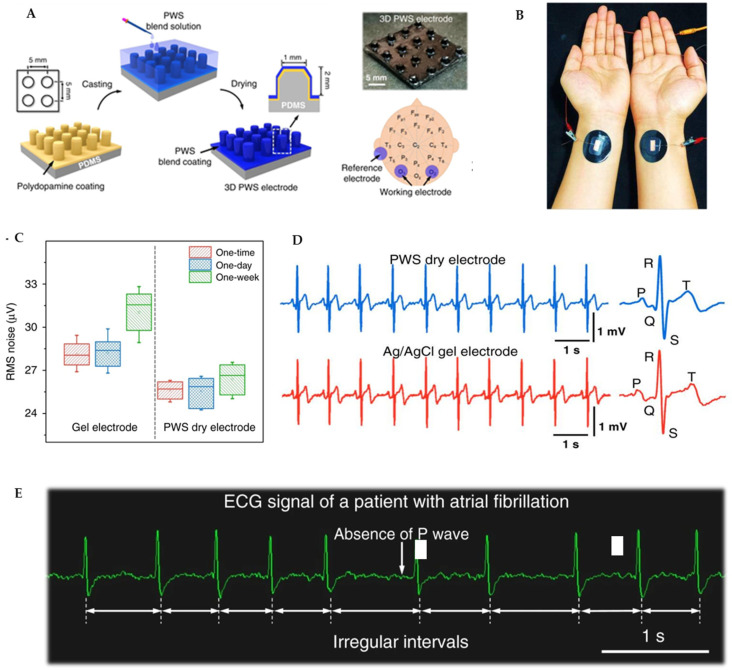
Studies with biosensing applications on electrophysiological signals: (**A**) Fully Organic Compliant Dry Electrodes Self-Adhesive to Skin for Long-Term Motion-Robust Epidermal Biopotential Monitoring (reproduced with permission from [147], copyright 2020, Nature)—Fabrication process of 3D PWS electrodes. (**B**) Photographs of PWS dry electrodes adhered to the wrist. The electrodes remained attached for 16 h and could be peeled off without causing skin irritation or redness. (**C**) RMS noise comparison between Ag/AgCl gel electrodes and PWS dry electrodes during ECG recording at one time point, after 1 day, and after 1 week. (**D**) ECG signal comparison using PWS dry electrodes versus commercial Ag/AgCl gel electrodes. (**E**) Clinical evaluation of PWS electrodes. ECG signals reveal variability in R-R intervals and absence of P-waves, indicative of atrial fibrillation.

Electrocardiography (ECG) measures the heart’s electrical activity and rhythm by detecting the signals produced with each heartbeat. This diagnostic tool helps identify and track various cardiac conditions. Traditional ECG monitoring typically relies on wet electrodes that use conductive gels to improve electrical conductivity between the skin and the electrode. However, these conventional approaches present limitations for extended monitoring periods, including skin irritation, signal degradation over time, and user discomfort. In response to these challenges, 3D printing technology helps to develop dry electrodes that offer better comfort while maintaining signal quality for long-term cardiac monitoring. Microneedle array electrodes represent a key advancement in dry electrode technology, as their design allows penetration into the epidermis, reducing the insulating effect of the stratum corneum and significantly lowering skin–electrode impedance. In a notable approach, 3D-printed PLA molds with cylindrical holes (≈1.5 mm diameter and 2 mm depth) were used to form micro-pillars by casting a PEDOT:PSS–WPU–D-sorbitol blend, curing at ~60 °C, and peeling off the patterned film [147] (Figure 6A–E). This fabrication yields electrodes with well-defined microstructures that achieve impedance levels comparable to or lower than traditional wet electrodes. The experimental results further confirm their superior ability to provide high-quality recordings of essential ECG components compared to conventional surface electrodes. In another study, Aloqalaa et al. evaluated the performance of 3D-printed ECG dry electrodes fabricated using four commercially available polylactic acid (PLA) conductive filaments [142]. The researchers designed, built, and tested electrodes specifically for acquiring ECG signals, comparing their performance based on signal-to-noise ratio (SNR) and their ability to accurately measure heart rate using the Pan–Tompkins algorithm. All the printed electrodes demonstrated acceptable efficiency, achieving SNR values equal to or exceeding 18.89 dB—a threshold that indicates sufficient signal quality for reliable heart rate measurement. In another study, the scientists developed a 3D-printed sensor capable of measuring both electroencephalogram (EEG) and electrocardiogram signals from zebrafish [141]. This approach demonstrated the potential for creating highly miniaturized biosensors suitable for small animal studies, which had previously been challenging due to size constraints. The work is particularly noteworthy considering the extremely small cranial area of zebrafish (approximately 2 mm × 2 mm), which requires exceptional precision in electrode placement and signal detection. Another recent study demonstrated the fabrication of wet electrodes using screen printing with silver nanowire ink, electrode gel, and gentle adhesive on flexible substrates [148]. The system integrated (1) multilayer screen-printed flexible electrodes with gel and biocompatible adhesive, (2) a compact 3D-printed wireless AoP readout connected via pogo pins, and (3) mobile/cloud-based analytics for continuous monitoring. Tested on 20 volunteers, the printed electrodes were rated significantly higher in comfort and ease of removal compared to commercial electrodes, highlighting the potential of 3D-printed, conformable electrodes for applications beyond EEG, including ECG. However, limitations remain, such as (1) performance degradation from gel drying (wet electrodes) or sweat/dirt accumulation (dry electrodes), (2) higher resistance in fully 3D-printed dry electrodes with conductive filaments, and (3) noise artifacts from EMG interference and baseline drift during motion [142]. Looking ahead, advances in hybrid nanomaterial inks (e.g., graphene and advanced polymer blends), flexible electronics for ultra-low impedance, and miniaturized, energy-efficient modules could enable true patch-like wearables. Moreover, direct printing onto garments or biocompatible hydrogels opens pathways toward next-generation “second-skin” or implantable monitoring systems [149].

Electromyography (EMG) is a diagnostic procedure that evaluates the health of muscles and the nerve cells (motor neurons) that control them. This technique involves recording and analyzing the electrical activity generated by skeletal muscles during contraction and relaxation. When muscles activate, they produce electrical signals that can be detected and measured, providing valuable information about muscle function and neuromuscular communication pathways. Traditional EMG measurements occur in clinical settings using either needle electrodes inserted directly into the muscle (invasive EMG) or surface electrodes placed on the skin overlying the muscle (non-invasive surface EMG or sEMG). Surface EMG has become particularly significant in the development of wearable biosensors as it allows for non-invasive, continuous monitoring of muscle activity. This approach forms the foundation for the expanding field of wearable EMG technology that enables monitoring outside clinical environments during everyday activities. Despite their promise, wearable EMG systems face several technical challenges. Maintaining stable skin–electrode contact during movement is particularly difficult, as poor contact can introduce motion artifacts and degrade signal quality [150]. To address this problem, Wan et al. developed a high-performance bioelectronic interface created using 3D printing of a novel poly (vinyl alcohol-formaldehyde) (PVAF)-PEDOT:PSS composite ink. This ink supports the precise printing of complex structures and yields a hydrogel interface with excellent conductivity, strong adhesion, and stable electrochemical properties (over 100 S/m). High adhesion (~31 kPa) and near-skin mechanical modulus prevent delamination and minimize motion-induced artifacts. The electrode comfortably conforms to dynamic skin movements, a distinct improvement over rigid or less-adhesive alternatives. When tested in EMG recording, the hydrogel electrodes outperform commercial types (such as Ag/AgCl) in signal stability, maintaining a high signal-to-noise ratio (>10 dB) under variable stresses and repetitive motion [151]. The integration of electrodes into textiles represents another significant advancement in wearable EMG technology. These “textrodes” can be incorporated directly into clothing, improving comfort and enabling unobtrusive monitoring during daily activities. Studies have shown that factors such as the electrode shape, fabric density, and applied pressure significantly affect signal quality [150]. For instance, wave-type embroidered electrodes have demonstrated greater morphological stability than circular designs, maintaining their shape even under strains up to 30% [152]. Additionally, research indicates that a minimum pressure of approximately 10 mmHg is necessary for textile electrode performance that is comparable to conventional Ag/AgCl electrodes [143]. Textrodes excel in wear comfort and enable continuous, unobtrusive monitoring in real-life settings. In a study, Li et al. developed a photocurable 3D printing approach for manufacturing graphene-enhanced polymer electrodes with programmable geometries. When incorporated into textile substrates, these printed nanocomposites demonstrated clinical-grade biosensing capabilities for both cardiac (ECG) and muscular (EMG) activity monitoring while maintaining comfort and durability for everyday use [153]. Another significant advancement in wearable EMG technology has been the development of ultra-low power systems that extend a battery’s life while maintaining high sampling rates. One notable approach uses two different frequency microcontroller clock sources and a ping-pong buffer memory architecture to achieve significant power savings [154]. These optimizations have resulted in power consumption reductions of up to 92.72% compared to commercial devices, with corresponding increases in battery life by more than nine times. Such advancements make continuous, long-term EMG monitoring increasingly practical for everyday use. Wearable EMG technologies are rapidly evolving with innovations in hydrogels, textiles, and nanocomposites that improve comfort, adhesion, and signal quality. However, challenges remain, including material instability from environmental changes, limited durability under mechanical stress, pressure-dependent signal quality in textiles, and unresolved concerns about biocompatibility, manufacturing scalability, and cost. Despite these hurdles, research is advancing toward hybrid and self-healing materials, multifunctional smart textiles, and fully integrated garments that combine sensing, electronics, and wireless connectivity.

Electroencephalography (EEG) measures the brain’s electrical activity through electrodes placed on the scalp, capturing postsynaptic potentials from cortical neurons. Traditional rigid EEG electrodes often cause discomfort, especially in hairy regions. Three-dimensional printing now enables flexible electrodes using materials like thermoplastic polyurethane (TPU), which adapt to scalp contours while maintaining conductivity. These non-conductive electrodes are coated with silver/silver-chloride (Ag/AgCl) to reduce contact impedance and noise, outperforming earlier rigid designs [155]. Few studies have also shown that 3D-printed fingered electrodes may be customized and that various electrode configurations can be employed for various individuals or for various head regions [140]. The flexible bases can part hair without snapping, enhancing long-term usability. Conductive filaments like Multi3D Electrifi allow fully 3D-printed dry electrodes, eliminating post-printing processing. These electrodes achieve contact impedances below 550 Ω (20 Hz–10 kHz) and successfully detect alpha wave fluctuations during eye-open/closed states [156]. Their customizable shapes cater to individual anatomies, addressing fit issues common in standardized designs. In another interesting study, researchers have transformed earbuds into EEG devices using 3D-printed stretchable sensors [157]. These conform to the ear’s dynamic structure, measuring brain activity and sweat lactate simultaneously. This dual sensing aids in epilepsy monitoring and performance tracking during exercise. The ear’s proximity to the brain and sweat glands makes it ideal for unobtrusive, high-fidelity data collection. These innovations open pathways for unobtrusive systems in healthcare, performance tracking, and even space or mobility-constrained settings. However, challenges remain in durability, signal stability under motion, scalability, and standardization, as customized geometries complicate universal benchmarking. Looking forward, hybrid sensing platforms, smart self-healing materials, and AI-guided optimization could enhance reliability and personalization, ultimately driving EEG technology toward robust, long-term, and user-friendly applications in both clinical and real-world environments. A compilation of studies demonstrating biosensing applications for electrophysiological signals with 3D-printed wearable biosensors is provided in Table 3.

### 5.2. Biochemical Signals

Traditional methods for measuring biochemical signals from the body have often relied on invasive techniques, primarily involving the extraction and analysis of blood samples. However, the limitations and inconvenience associated with these procedures spurred the development of non-invasive approaches utilizing alternative biofluids like saliva, tear fluid, and sweat. The ability to access biochemical information through these less intrusive means has paved the way for continuous and non-invasive monitoring, a capability that has significantly fueled the burgeoning field of wearable technology, enabling real-time health and wellness tracking.

**Figure 7:**
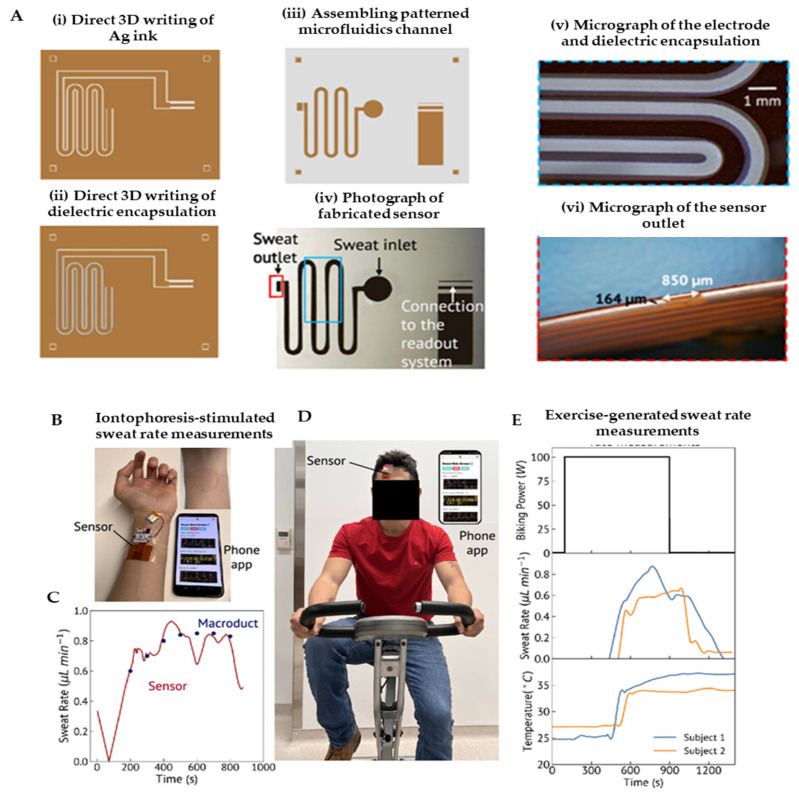
Studies with biosensing applications on biochemical signals: (**A**) Printed Wearable Sweat Rate Sensor for Continuous In Situ Perspiration Measurement (reproduced with permission from [145], copyright 2025, Nature)— The fabrication process involves: (**i**) direct 3D writing of two parallel Ag electrodes; (**ii**) direct 3D writing of dielectric encapsulation; (**iii**) integration of a double-sided microfluidic tape, patterned with a desktop cutter machine, onto the encapsulated Ag electrodes; (**iv**) photographs of a completed SR sensor patch highlighting the sweat inlet, outlet, and connectors for readout electronics; (**v**) micrographs of the dielectric encapsulation around the metal electrodes (scale bar: 1 mm); and (**vi**) micrographs of the sensor outlet, which has a thickness of 164 μm and a width of 850 μm. (**B**) On-body SR sensing. A photograph shows the sensor worn on the left forearm along with a snapshot of the mobile application. The inset highlights the iontophoresis area. The SR values obtained from the rate sensor match those measured using the Macroduct sweat collection system. (**C**) A photograph shows the sensor worn on the forehead while the subject exercises on a stationary bike, accompanied by a snapshot of the mobile application. (**D**) Power output of the stationary bike during exercise and on-body SR measurements from two subjects. Sweating begins after a certain time, and SR decreases once the bike power is reduced to zero. (**E**) On-body temperature measurements. Both subjects show an increase in temperature at the onset of sweating.

Sweat-based biosensors have emerged as a pivotal tool for non-invasive, real-time health monitoring, leveraging sweat’s rich content of biomarkers like electrolytes, metabolites, and hormones. These biosensors use electrochemical platforms, such as potentiometric and amperometric sensors, to detect key indicators like lactate, glucose, and cortisol, offering insights into physiological states, including stress and fatigue [174]. Additionally, electrolytes such as chloride and copper, as well as pH levels in sweat, have been successfully measured using 3D-printed microfluidic systems with integrated microcuvettes [175]. Islam et al. created a flexible sweat rate sensor utilizing 3D-printed microfluidic channels combined with capacitive electrodes on a flexible substrate (Figure 7A–E). The device achieved a high sensitivity of 0.01 μL/min, enabling real-time hydration tracking that was seamlessly integrated with a mobile application for data visualization. Its major advantages include the capacity for continuous monitoring and its high sensitivity, making it an ideal tool for athletes and workers in labor-intensive environments. A key limitation is that the sensor primarily measures the sweat volume and flow rate without providing data on biochemical composition; furthermore, its performance under fluctuating ambient humidity conditions requires further validation [145]. Complementing this, Liu et al. designed an eco-friendly chromic device, fabricating a 3D-printed flexible patch that incorporated materials that visually change color in response to UV exposure, temperature, and sweat pH. The device produced stable visible readouts and was lauded for its fully recyclable and environmentally conscious design. Its primary advantages stem from the complete absence of electronic circuitry, which drastically reduces both cost and electronic waste, while also enabling intuitive visual interpretation. However, a significant limitation is its inherent lack of quantitative precision, as interpreting color shifts can be subjective, and the platform is not multiplexed for the detection of numerous analytes simultaneously [176]. Koukouviti et al. developed an enzyme-free glucose sensor featuring a 3D-printed PLA electrode modified with an Fe(III) cluster for direct voltammetric detection [177]. The sensor demonstrated selective glucose quantification within the acidic environment of sweat (pH~4–6) and maintained excellent stability by eliminating reliance on fragile biological enzymes. Its key advantages include overcoming a major hurdle in wearable biosensing—enzyme instability—while utilizing a low-cost PLA substrate. A primary limitation is that the study exclusively focused on glucose, leaving its performance and potential cross-reactivity in the complex, multi-analyte milieu of real sweat unclear. The study has a significant potential in expanding this robust enzyme-free platform to detect other biomarkers like lactate, cortisol, or uric acid, thereby enabling the development of comprehensive multi-analyte sensing devices. In another study, Mi et al. combined MOF-derived porous carbon nanorods with a 3D-printed microfluidic chip to enable simultaneous monitoring of uric acid and potassium ions in sweat, showcasing the platform’s multiplexing potential and high analytical performance [178]. Across recent studies, 3D printing is demonstrably democratizing biosensor fabrication by offering highly customizable, scalable, and low-cost production methods. However, key limitations persist, including a lack of standardization in biomarker thresholds, susceptibility to environmental interference like variable sweat rates and pH, and a general inability to effectively multiplex beyond one or two analytes. Many devices also remain in early validation stages, tested under controlled conditions rather than during prolonged real-life use, and some still depend on external readers. Future directions are consequently focused on developing fully integrated multiplex systems that combine the sweat rate, metabolites, and electrolytes, leveraging AI for data interpretation and personalized calibration to individual physiology.

Saliva-based biosensors are gaining significant traction as non-invasive, point-of-care diagnostic tools capable of detecting a wide array of biomarkers, from metabolic and infectious diseases to stress and hormonal imbalances. These sensors utilize the rich biochemical milieu of saliva—including electrolytes, enzymes, nucleic acids, and metabolites—to enable real-time health monitoring with minimal patient discomfort. Electrochemical biosensors, particularly organic electrochemical transistors (OECTs) and aptamer-based platforms, offer high sensitivity and have been successfully deployed to detect analytes like glucose, uric acid, and SARS-CoV-2, demonstrating potential as alternatives to conventional blood-based diagnostics [179,180]. Several 3D printing methods have demonstrated utility in creating saliva-based sensors. Fused deposition modeling (FDM) has emerged as a versatile approach, enabling the production of highly customizable and complex geometries (e.g., microfluidic channels and multi-analyte systems) that are essential for advanced clinical diagnostics. A significant trend is the development of bespoke conductive filaments (e.g., PLA composites with graphene, carbon nanotubes, or metal nanoparticles), which are tailored to enhance electrochemical performance by optimizing conductivity and biocompatibility, surpassing the limitations of commercial filaments like Black Magic^®^ or Proto-Pasta^®^ that often contain impurities affecting sensor reliability [118]. Wrobel Von Zuben et al. developed a 3D-printed amperometric sensor using a polylactic acid (PLA) thermoplastic composite infused with graphene flakes for the enzyme-free detection of ethanol in saliva samples. The fused deposition modeling (FDM) technique enabled the fabrication of electrodes that demonstrated strong sensitivity and reproducible signals, leveraging the conductive properties of graphene within the PLA matrix to facilitate efficient electron transfer during ethanol oxidation. A key advantage of this non-enzymatic design is its avoidance of the stability issues commonly associated with biologically modified electrodes, enhancing durability and reducing operational complexities. However, the study noted that the dispersion quality of graphene flakes within the PLA filament significantly influenced electrical conductivity and sensor-to-sensor reproducibility, highlighting a critical manufacturing challenge that requires further optimization [181]. These composite materials combine the printability of thermoplastics with the electrical conductivity and sensing capabilities of nanomaterials. Sunil et al. developed a sophisticated 3D-printed microfluidic SERS biosensor platform incorporating Cu@Ag core–shell nanoparticle-decorated carbon nanofibers (Cu@Ag/CNFs) for the non-invasive, label-free detection of salivary biomarkers associated with oral cancer [182]. The 3D-printed system demonstrated strong Raman enhancement, enabling high-sensitivity identification of biomarkers in both simulated and clinical saliva samples with a high signal-to-noise ratio. The advantages of the system include the platform’s capacity for high-throughput screening and its integration with AI-assisted spectral analysis, which enhances diagnostic accuracy by automating the interpretation of complex Raman spectra. However, challenges remain in the fabrication complexity and batch-to-batch reproducibility of nanoparticle deposition, while the cost of the device currently exceeds that of conventional electrochemical biosensors. Another recent development is a 3D bioprinted hydrogel sensor for salivary pH detection, employing a sodium alginate–polyvinylpyrrolidone matrix and bromothymol blue to provide a robust colorimetric response in the pH range of 3.5 to 9.0 [146]. This design supports easy visualization and digital analysis via RGB component extraction, making it suitable for wearable intraoral applications. These innovations underscore the versatility of 3D printing for tailoring sensor architecture, improving sensitivity, and enhancing user compliance. However, key limitations remain, such as biofouling from salivary mucins and enzymes, variability between individuals requiring personalized calibration, and a general lack of multi-analyte capability in current devices. Looking forward, the integration of hybrid detection modalities, machine learning for biomarker pattern recognition—as seen in AI-aided SERS platforms—and the development of self-powered intraoral wearables present promising pathways toward standardized, energy-autonomous, and clinically validated salivary diagnostic systems for continuous health monitoring.

Another emerging transformative tool in non-invasive diagnostics is tear-based biosensors. These biosensors harness ocular platforms, particularly smart contact lenses and eye patches, to continuously monitor biomarkers such as glucose, lactate, electrolytes, pH, and proteins. Although it is still an emerging field, 3D-printed wearable tear-based biosensors are showing immense promise in non-invasive, real-time health monitoring, particularly via smart ocular platforms. While specific studies directly focusing on 3D-printed tear biosensors are limited, several reviews and technology demonstrations indicate a strong potential for adapting 3D-printed methods for tear analysis. Kalkal et al. highlight advancements in the 3D printing of biosensors for wearable healthcare, noting the feasibility of miniaturized devices for ocular use through vat photopolymerization and material extrusion—techniques that are compatible with soft bio-functional materials that are suitable for tear interfaces [183]. Rachim and Park expand on this by discussing in situ 3D printing directly onto curved, non-planar surfaces, like the human eye, enabling personalized, bio-integrated sensors for tear fluid detection [184]. Ho et al. demonstrated 3D-printed sugar-based scaffolds for wearable sensors, which offer high flexibility and biocompatibility—essential traits for ocular applications—though these are not yet specifically applied to tear fluid [110]. The integration of piezoelectric elements to power 3D-printed sensors, as shown by Sobianin et al., also hints at future self-powered tear biosensing platforms [144]. While 3D-printed tear biosensors are still largely conceptual, these foundational studies support their eventual realization, potentially via soft contact lenses or eye patches tailored through additive manufacturing. Table 4 summarizes studies that explore biosensing applications of 3D-printed wearable biosensors for biochemical signal monitoring.

### 5.3. Vascular System Dynamics

Vascular dynamics refer to the complex patterns of blood flow through the body’s circulatory system. These dynamics are governed by fluid–structure interactions between blood and vessel walls, creating measurable waveforms that contain valuable diagnostic information. Continuous monitoring of these dynamics enables early detection and prevention of cardiovascular diseases, which remain a leading cause of mortality worldwide.

**Figure 8:**
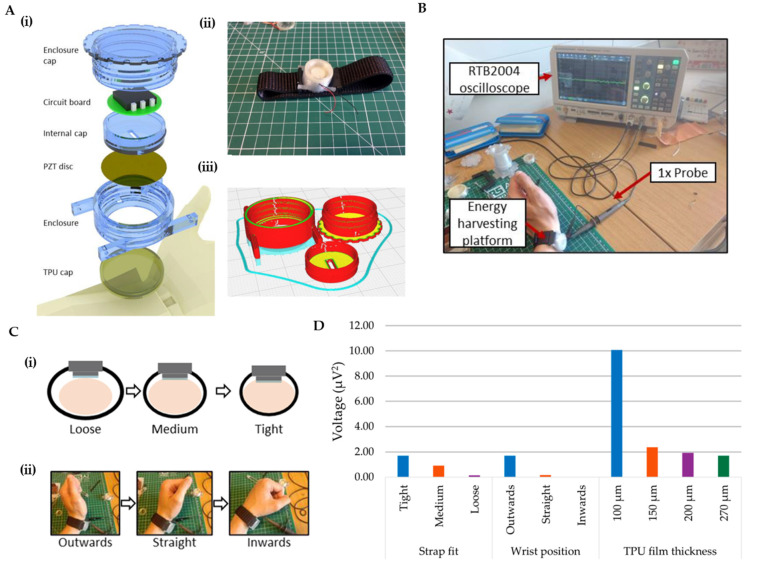
Studies with biosensing applications on vascular dynamics. (**A**) A 3D-Printed Piezoelectric Microdevice for Human Energy Harvesting for Wearable Biosensors (reproduced with permission from [144], copyright 2024, MDPI)—(**i**) schematic of the energy-harvesting platform assembly; (**ii**) photograph of the manufactured prototype; (**iii**) sliced PET components.; (**B**) experimental setup; (**C**) (**i**) Strap fit configurations; (**ii**) wrist positions evaluated; (**D**) comparative results obtained from the parametric study.

Multiple sensing mechanisms have been employed in 3D-printed wearable devices for detecting vascular-related biosignals. Pulse-wave monitoring represents one of the most common applications for 3D-printed wearable vascular sensors. Self-powered, high-sensitivity printed e-tattoo sensors have been developed for unobtrusive arterial pulse-wave monitoring [206]. Continuous blood pressure monitoring is crucial for real-time assessment and early prevention of cardiovascular diseases. Wearable continuous blood pressure monitoring devices based on pulse-wave transit time have received significant attention due to their excellent dynamic response characteristics and high accuracy [207]. These systems typically combine photoplethysmogram (PPG) and ECG measurements to calculate the transit time between arterial sites, which correlates with blood pressure. Numerous clinical and consumer devices measure the PPG signal, which has become a useful tool for determining the age and function of the arteries. Variations in blood pressure, atherosclerosis, changes in arterial stiffness, and natural vascular aging all affect the PPG pulse’s waveform and timing [207,208]. Piezoelectric sensing technologies have been incorporated into 3D-printed wearable devices to detect mechanical vibrations originating from arterial pulsations (Figure 8A–D). These components can not only sense vascular activity but also harvest energy from arterial pulsations, potentially enabling self-powered operation. This dual functionality makes piezoelectric-based sensors particularly attractive for long-term monitoring applications [144]. Three-dimensional-printed wearable ring sensors incorporating MEMS piezo-resistive pressure sensors have demonstrated accurate monitoring of real-time human blood pressure pulse waveform as an indicator for cardiovascular conditions [209]. Their ring achieved an accurate heart rate (HR) and HRV readings that are comparable to a clinical ECG strap and could track the entire blood pressure pulse’s waveform over long-term wear. Because it captures the waveform shape, it has potential for early hypertension screening: the authors noted the ring could detect subtle changes or abnormalities in the waveform, enabling early diagnosis. Phonoangiography (PAG) represents another valuable sensing approach that captures acoustic signals generated by blood flow through vessels. Custom-designed 3D-printed wearable devices combining PAG and PPG techniques have shown promise for the early and accurate detection of arteriovenous access (AVA) stenosis in hemodialysis patients. Research has demonstrated that after percutaneous transluminal angioplasty (PTA), the amplitudes of both PAG and PPG signals increased in patients, corresponding with improved blood flow [210].

The materials used in 3D-printed vascular monitoring devices significantly influence their performance, comfort, and durability. Conductive hydrogels have emerged as promising materials for 3D-printed wearable sensors due to their excellent biocompatibility, flexibility, and electrical conductivity [211]. These materials can be 3D-printed while maintaining their conductive properties, making them ideal for direct skin contact applications like pulse sensing. Elastic thermoplastic polyurethane (TPU) films are frequently incorporated to provide better skin adherence, protect sensitive electronic components, and electrically isolate the device from the human body. In one notable application, a TPU film forms an air chamber between the skin and the piezoelectric disc electrode, improving adsorption to the skin while preventing damage to the piezoelectric component [144]. Triboelectric materials have also been utilized in wearable sensors for pulse wave monitoring. Wearable triboelectric nanogenerator (TENG)-based sensors offer compelling advantages, including self-powered operation, lightweight construction, and superior sensitivity [212]. Table 5 presents a list of studies on biosensing applications for signals from vascular dynamics using 3D-printed wearable biosensors. A compilation of studies demonstrating biosensing applications due to body mechanical deformation (strain sensor), touch sense (tactile sensor), and other miscellaneous physiological signals with 3D-printed wearable biosensors is provided in Table 6.

## 6. Implantable Devices by 3D Printing Technology

Implantable biosensing devices represent a frontier in healthcare monitoring, offering direct access to physiological parameters and biomolecular markers within the body. These devices enable continuous monitoring of internal biological processes that cannot be accessed through non-invasive means, providing critical data for disease diagnosis and management. Recent advances in 3D printing have significantly expanded the possibilities for implantable sensor design, allowing for the creation of biocompatible structures that can interface effectively with surrounding tissues while minimizing foreign body responses. Three-dimensional printing methods, such as fused deposition modeling (FDM), stereolithography (SLA), selective laser melting (SLM), and direct ink writing (DIW), can fabricate structures from metals, polymers, hydrogels, or composites with customized shape and porosity. For example, FDM and SLA are widely used for electrode scaffolds and microfluidic chips, while DIW can extrude viscous inks (e.g., graphene or conductive polymers) for soft or tissue-like probes [248]. A notable innovation in this field is the development of 3D-printed liquid-core hydrogel platforms for encapsulating single-walled carbon nanotube (SWNT) sensors, which can detect important signaling molecules such as nitric oxide (NO) and hydrogen peroxide (H_2_O_2_) [249]. This approach addresses previous challenges related to sensor stability and compatibility with biological environments, providing a promising solution for long-term implantable molecular sensing.

Implantable biosensors typically rely on electrochemical or optical transduction. Electrochemical modes include amperometric (enzymatic or redox sensors), potentiometric (ion- or pH-sensitive), and impedance-based detectors [248]. For instance, implanted amperometric electrodes can monitor local neurotransmitter or metabolite levels in real time [250]. Optical approaches (fluorescence or luminescence) have also been integrated into 3D-printed scaffolds for reporter-based sensing. Other modalities (capacitive, piezoelectric, and thermal) are more common in mechanical/strain-sensing implants [248].

### 6.1. Implantable Biosensors for Neurological Applications

Neurological disorders represent a critical application area for implantable biosensors, as continuous monitoring of neural activity and neurotransmitters can provide invaluable insights for diagnosis and treatment. Momin et al. created a 3D-printed flexible neural probe with a porous, tissue-like silicone–carbon composite structure [251]. Their DIW device matched the brain’s compliance and achieved low impedance for high-fidelity recordings at the single-neuron level. Other groups have DIW-printed electrodes from graphene or PEDOT:PSS [252]. Shao et al. reported 3D-printed carbon nanoneedles (via photopolymerization and pyrolysis) that were sharp enough to measure dopamine release in a Drosophila nerve cord. Graphene-based 3D-printed sensors are especially promising for Parkinson’s monitoring. Dopamine depletion is a hallmark of Parkinson’s disease, so that implantable dopamine sensors could aid early diagnosis or therapy. Shin et al. created a minimally invasive system using a graphene electrode array in very encouraging research. This is the first integrated system to concurrently demonstrate biocompatibility, wearability, removability, target specificity, and wireless control [253]. Animal models of Parkinson’s disease have shown that cortical motor surface stimulation normalizes brain waves and restores motor function, which corresponds to potentiated synaptic responses. Moreover, the overexpression of the D5 dopamine receptor (D5R, Drd5) and metabotropic glutamate receptor 5 (mGluR5, Grm5) genes in the glutamatergic synapse is linked to these alterations. The wireless capabilities of this neural implant enable both real-time diagnostics and targeted therapeutics, suggesting significant potential for clinical applications in Parkinson’s disease treatment. A recent review notes extensive development of graphene-modified electrodes for dopamine detection [250]. Graphene can be formulated into printable inks or composites: for instance, a graphene/PLA filament was 3D-printed into a microneedle-like electrode array for ex vivo dopamine sensing. In general, such electrochemical implants would use chronoamperometry or voltammetry to detect neural transmitters or metabolites with high sensitivity and spatial resolution. These systems must also integrate with telemetry or onboard electronics to transmit data out of the body.

### 6.2. Bone Regeneration and Orthopedic Sensors

Three-dimensional printing has been widely applied to bone repair scaffolds and implants. Smart bone scaffolds can combine biocompatible matrices (e.g., titanium, ceramics, or polymers) with sensing functionality. For instance, Huang et al. demonstrated a 3D-printed porous scaffold of carboxymethyl chitosan reinforced with 0.5% (*w*/*v*) carboxylated carbon nanotubes (CNT) for bone defects [254]. This CNT/CMC scaffold was electrically conductive and exhibited “electrochemical responsiveness”: cyclic voltammetry and impedance spectra changed sensitively as osteogenic cells differentiated, thus reporting on new bone formation. Notably, the CNTs also enhanced scaffold strength and stimulated stem cell osteogenesis. This is an example of a biosensing scaffold—it does not have discrete electronics, but its material itself acts as a sensor. Other approaches embed miniature sensors into orthopedic implants. For example, Lavdas et al. designed a titanium orthopedic spacer with a cavity housing a temperature sensor and wireless telemetry for monitoring post-operative infection in knee arthroplasty [255]. The sensor electronics were encapsulated in a 3D-printed Ti case within the bone cement. Similarly, Feynman-track strain gauges and piezoelectric elements have been integrated into 3D-printed knee or spine implants for load sensing.

A clinical case report describes the use of a 3D-printed titanium mesh implant with a plate construct for managing critical-size bone defects in distal tibial open wounds [256]. The porotic nature of the mesh facilitated bone ingrowth, with CT scans at 1.5 years post-surgery confirming good bone integration and restored ambulation. This case demonstrates how 3D-printed implants can effectively integrate with biological tissues, suggesting potential for incorporating biosensing elements that could monitor bone healing processes in real-time. However, as noted by recent reviews, electrochemical sensors (chemical or biochemical) are still rare in orthopedics [248]. When used, implantable sensors may target pH (to detect inflammation/infection), phosphate or cytokine levels, or pressure (strain) at the bone–implant interface. In all cases, biocompatibility and long-term stability are major concerns—implants must function reliably for months or years without leaching or significant drift.

### 6.3. Tumor and Cancer Biomarker Sensors

Implantable sensors for oncology are an emerging frontier. The goal is to measure tumor-specific biomarkers (proteins, DNA, metabolites) in situ, enabling personalized therapy monitoring. One concept is an implantable electrochemical aptasensor: for example, a printed graphene-based electrode functionalized with a DNA aptamer for cancer antigen (like HER2 or MUC1) could be inserted near a tumor. Similarly, 3D-printed microdialysis probes or hydrogel reservoirs can continuously sample interstitial tumor fluid, feeding analytes to an on-chip sensor [248]. In general, electrochemical biosensors are well-suited to detect proteins, nucleic acids, or small molecules.

### 6.4. Biocompatibility, Stability, and Regulatory Considerations

Although additive manufacturing supports patient-specific geometries (e.g., matching bone defect topology) and rapid prototyping of multi-material systems; however, printing implants mandates rigorous material control and post-processing (such as cleaning, sterilization, and curing) to meet the biocompatibility and mechanical requirements [248]. Any implantable device must use biocompatible materials (ISO 10993 compliant) and be thoroughly sterilizable [257]. Metals (Ti, CoCr) and ceramics (hydroxyapatite, beta-TCP) used in bone implants have well-known biocompatibility. Polymers like medical-grade PLA/PCL, PEEK, parylene, polyimide, or silicone are also common. Three-dimensional printable hydrogels based on conducting polymers have emerged as promising materials for creating implantable bioelectronics with tissue-like mechanical compliance and robust electrochemical properties [258]. These materials offer advantages such as Young’s modulus values around 650 kPa (like soft tissues), strong bioadhesion properties (interfacial toughness of 200 J m^−2^ and shear strength of 120 kPa), and tunable electrical properties.

Longevity is a key challenge: implant sensors must operate for months or years without significant drift. Chronic implantation leads to protein adsorption and encapsulation by fibrotic tissue, which can degrade sensor signals. Strategies to mitigate fouling include anti-biofouling coatings (PEG or zwitterionic materials) or self-cleaning surfaces. Recent research has demonstrated promising advances in sensor longevity, with some 3D-printed hydrogel-encapsulated sensors maintaining stable performance for extended periods. For instance, (AT)_15_-wrapped-SWNT NO sensors encapsulated in 3D-printed self-healing hydrogels have shown a negligible decrease in fluorescence intensity after 90 days at 37 °C, with statistical analyses indicating that the change in intensity was not significant [249].

Reliability testing in vitro and in vivo (accelerated aging, bioreactor flow) is essential. For example, carbon electrodes may suffer surface oxidation; polymers may hydrolyze, and printed layers may delaminate. Thus, multi-month animal studies and benchtop soak tests are typically required before clinical use. In recent work, an electrophysiological study in rat heart models has demonstrated the capability of 3D-printed hydrogel bioelectronics to establish conformal interfaces with dynamic organs, enabling long-term and high-precision spatiotemporal epicardial monitoring [258]. These developments highlight the potential of 3D-printed implantable biosensors for chronic disease monitoring and management applications that require stable, long-term performance.

Three-dimensional-printed implantable sensors fall under medical device regulations (e.g., FDA Class III or EU MDR, depending on the risk). Regulatory bodies require demonstration of safety, efficacy, and manufacturing control. This includes adherence to ISO standards (biocompatibility ISO 10993, sterilization ISO 11135/11737, electrical safety ISO 60601) and good manufacturing practices [257]. For additive-manufactured devices, the FDA has issued guidance (2017) emphasizing the validation of printing processes, material traceability, and post-process quality control [259]. Custom or patient-specific implants must follow stringent design controls. In practice, this means every novel printed implantable sensor needs bench testing (electrical/analytical performance), biocompatibility studies, and often animal studies (to show no toxicity or adverse tissue response) before human trials.

Despite the hurdles, several 3D-printed implants (mostly orthopedic scaffolds and dental implants) have already reached clinical use, indicating feasibility [260]. The field of 3D-printed biosensors is younger, but momentum is growing. Combining additive manufacturing with advances in flexible electronics and nanomaterials holds promise for next-generation “smart implants” that monitor health from within the body. In conclusion, 3D printing provides unique advantages (such as customization, integration, and speed) for implantable biosensors, and recent studies have demonstrated prototypes in bone, neural, and microfluidic domains [250,254,261].

## 7. Challenges and Future Perspectives

Recent advancements in 3D printing technologies have revolutionized the development of biosensing devices, offering unprecedented opportunities for personalized health monitoring through wearable and implantable sensors. These innovations enable continuous, real-time monitoring of physiological and biochemical parameters, potentially transforming disease diagnosis and management. Despite these advances, numerous challenges persist regarding material selection, printing resolution, biocompatibility, and long-term reliability.

### 7.1. Material Challenges in 3D Printing of Biosensors

Material selection represents a fundamental challenge in 3D printing of biosensing devices, as the materials must simultaneously satisfy requirements for printability, sensor functionality, and biocompatibility. Traditional 3D printing materials often lack the necessary electrical, optical, or chemical properties required for effective biosensing applications. For instance, stereolithographic (SLA) photopolymers typically yield parts with low mechanical compliance, which are unsuitable for applications requiring tissue-like flexibility. Researchers have addressed this challenge by developing tunable resins with polydimethylsiloxane (PDMS)-like elastic modulus for stereolithographic 3D printing, enabling the creation of more compliant structures for biosensing applications [262]. Additionally, material compatibility with different 3D printing methods presents a significant bottleneck in the development of functional biosensing devices [123]. Each printing technique (extrusion-based, stereolithographic, inkjet, etc.) imposes specific requirements on material viscosity, curing mechanisms, and thermal properties, limiting the range of viable materials for specific biosensor designs.

The incorporation of nanomaterials into printable formulations offers enhanced functionality but introduces additional challenges related to dispersion stability and printing reliability. Nanocomposites incorporated into 3D printing for biosensors include carbon nanotubes, metal nanoparticles, and conductive polymers, which can significantly enhance sensor sensitivity and selectivity [107]. However, achieving uniform dispersion of these nanomaterials within printing resins or inks without agglomeration remains challenging, particularly for high-aspect-ratio nanomaterials such as carbon nanotubes. The tendency of nanomaterials to settle or aggregate during the printing process can lead to inconsistencies in sensor performance across different parts of the printed structure. Furthermore, the incorporation of nanomaterials can alter the rheological properties of printing inks, potentially compromising printability and resolution [263]. These challenges necessitate careful formulation development and process optimization to ensure reliable production of functional nanocomposite-enhanced biosensors.

The biocompatibility and biofunctionality of printed materials present another layer of complexity, particularly for implantable biosensing applications. Materials must not only be non-toxic and non-immunogenic but also maintain their functional properties in the biological environment over extended periods. The development of biomaterials capable of withstanding a harsh physiological environment while maintaining sensing capabilities requires careful consideration of surface chemistry, degradation behavior, and protein adsorption characteristics [123]. Additionally, the potential release of unreacted monomers, photoinitiators, or degradation products from printed materials can cause adverse biological responses, necessitating thorough biocompatibility evaluations. Recent advances in this area include the development of 3D printable hydrogels with self-healing properties and robust bioadhesion, which can establish stable interfaces with dynamic biological tissues [258]. Despite these advances, the limited range of biocompatible materials suitable for different 3D printing technologies continues to constrain the design space for wearable and implantable biosensing devices.

### 7.2. Technical Challenges in 3D Printing of Biosensors

Printing resolution remains a significant technical challenge in the fabrication of biosensing devices, particularly for applications requiring microscale or nanoscale features. While commercial 3D printers have made remarkable progress in resolution capabilities, there still exists a substantial gap between the resolution achievable with current 3D printing technologies and the feature sizes required for optimal biosensor performance [123]. This limitation is particularly pronounced for sensors targeting the detection of small biomolecules or requiring high-density electrode arrays. High-resolution techniques such as two-photon polymerization can achieve sub-micron features but suffer from limited throughput and material compatibility issues.

Multi-material printing capability is essential for creating integrated biosensing systems, but it introduces numerous technical complexities. Biosensors typically require the integration of materials with disparate properties-such as conductive elements for signal transduction, flexible substrates for comfort and conformability, and bioactive components for molecular recognition [107]. Researchers have developed approaches such as “Pause-Print” protocols (3P-printing) to fabricate high-resolution multi-material parts with desktop SLA printers without requiring post-assembly [261]. However, challenges persist related to material compatibility, adhesion between different materials, and preventing cross-contamination during the printing process. The integration of electronic components and circuitry with 3D-printed structures presents another layer of technical complexity. Most biosensors require not only the sensing element itself but also associated electronics for signal conditioning, processing, and transmission [264]. Recent innovations have explored approaches for printing electronic circuits directly within 3D structures using conductive inks or embedding pre-fabricated electronic components during the printing process [258]. However, these approaches face challenges related to ensuring proper electrical connectivity, preventing thermal damage to electronic components during printing, and maintaining mechanical integrity at the interface between rigid electronics and potentially flexible printed substrates.

### 7.3. Operational Challenges

Sensor performance and reliability under real-world conditions represent significant operational challenges for 3D-printed biosensing devices. Many biosensors demonstrate excellent performance in controlled laboratory environments but fail to maintain consistent functionality when exposed to the complex, dynamic conditions of actual use [264]. For wearable sensors, factors such as motion artifacts, skin perspiration, temperature fluctuations, and mechanical deformation during body movement can significantly impact sensor readings and reliability [107]. Implantable sensors face even more challenging conditions, including protein biofouling, immune responses, tissue encapsulation, and potential degradation in the physiological environment [249]. These challenges are compounded for 3D-printed sensors, as the layer-by-layer fabrication process can introduce structural heterogeneities that compromise mechanical integrity and sensing performance under stress.

Power management presents a critical operational challenge, particularly for wireless and implantable biosensing devices. The continuous operation of sensors for real-time health monitoring demands efficient power utilization strategies to extend device lifespan between charging cycles or battery replacements [264]. For implantable sensors, battery replacement typically requires invasive procedures, making long-term power sustainability especially crucial. Traditional approaches to power miniaturized sensors include small batteries, wireless power transfer, and energy harvesting from the environment or body [265]. However, the integration of these power solutions with 3D-printed structures introduces additional design and fabrication complexities. Recent research has explored the potential of 3D printing to create energy storage systems directly within sensor structures, as well as the development of self-powered devices that can harvest energy from physiological processes or environmental sources [123]. Despite these advances, achieving a balance between power consumption, sensor performance, and device size remains challenging, particularly for continuous monitoring applications that require frequent data acquisition and transmission.

### 7.4. Future Perspectives

The integration of artificial intelligence and machine learning with 3D-printed biosensing devices represents a promising frontier for enhancing sensor capabilities and clinical utility. As biosensors continue to generate increasingly complex and voluminous data, AI algorithms can help identify subtle patterns and correlations that might indicate early disease onset or treatment efficacy [264]. Machine learning approaches can also compensate for sensor limitations by filtering out noise, correcting drift, and improving overall accuracy through adaptive calibration techniques. Furthermore, AI-enabled personalization of sensor systems could allow for adapting detection parameters based on individual physiological baselines and health histories, leading to more meaningful and actionable health insights [266]. The combination of 3D printing’s customization capabilities with AI’s analytical power could enable the development of highly personalized biosensing systems tailored to individual patient needs, anatomical considerations, and specific health monitoring requirements. This convergence of technologies is likely to significantly enhance the clinical value of biosensing devices while expanding their applications across diverse healthcare settings.

Emerging materials and printing technologies are poised to revolutionize the capabilities of wearable and implantable biosensors. Advanced bioinks incorporating stimuli-responsive polymers, self-healing materials, and biomimetic structures are being developed to improve sensor biocompatibility, longevity, and functionality in complex biological environments [258]. For instance, 3D-printable hydrogels with self-healing properties and strong bioadhesion have demonstrated the ability to maintain stable interfaces with dynamic organs, enabling long-term electrophysiological monitoring. Novel 4D printing approaches, where printed structures can change shape or properties in response to environmental stimuli, offer exciting possibilities for creating adaptive biosensors that can respond to physiological changes or optimize their positioning within tissues [123]. Additionally, advances in high-resolution printing technologies, such as two-photon polymerization and microsterolithography, are enabling the fabrication of biosensors with nanoscale features, potentially leading to significant improvements in sensitivity, specificity, and miniaturization [263]. These materials and technological innovations are expanding the design space for biosensing devices while addressing many of the current limitations related to biocompatibility, durability, and performance.

The clinical translation and commercialization of 3D-printed biosensing technologies represent critical steps toward realizing their full potential in healthcare. While numerous innovative 3D-printed biosensors have been demonstrated in research settings, relatively few have progressed to clinical validation and commercial availability [107]. Bridging this translational gap requires addressing several key challenges, including scaling up manufacturing processes while maintaining quality and consistency, establishing regulatory pathways for novel device approval, and demonstrating clear clinical benefits and cost-effectiveness compared to existing monitoring approaches [264]. Additionally, developing sustainable business models that balance device affordability with manufacturing costs will be essential for widespread adoption, particularly in resource-limited healthcare settings [266]. Despite these challenges, the unique advantages offered by 3D-printed biosensing devices—including customization, rapid prototyping, and potential for point-of-care manufacturing—position them favorably for future clinical integration, potentially transforming approaches to disease monitoring, management, and personalized healthcare delivery.

## 8. Conclusions

The evolution of biosensor technology has traversed a remarkable journey from Clark’s pioneering glucose electrode in 1962 to today’s sophisticated wearable and implantable devices. This review has highlighted how 3D printing technology is revolutionizing biosensor fabrication, addressing critical limitations of conventional manufacturing methods. The unique capabilities of additive manufacturing—including design freedom, cost-effectiveness, rapid prototyping, and material versatility—have enabled the production of complex, miniaturized biosensing platforms that were previously unattainable. Particularly significant is 3D printing’s ability to create customized, patient-specific devices with intricate geometries while integrating multiple materials and sensing modalities into unified structures. These advantages have accelerated the development of flexible, stretchable, and lightweight biosensors that conform seamlessly to biological surfaces, enhancing both comfort and performance. As we look toward the future, the convergence of 3D printing with advanced nanomaterials, multifunctional bioinks, and AI-powered analytics promises to further transform biosensor technology. While challenges remain in material optimization, durability, and regulatory standardization, the trajectory is clear: 3D-printed biosensors are poised to dramatically expand point-of-care diagnostics, enable continuous health monitoring, and ultimately drive a new paradigm of personalized, proactive healthcare with profound implications for both clinical practice and patient outcomes.

## Figures and Tables

**Table 1:** Biosensor: Common targets, detection strategies, and applications.

Analyte	Bioreceptor	Detection Method (Transducer)	Applications	Reference
Glucose	Glucose oxidase, Glucose dehydrogenase	Electrochemical (Amperometric, Potentiometric), Optical	Diabetes monitoring, metabolic studies	[4]
Lactose	β-Galactosidase, Lactose oxidase	Electrochemical (Amperometric), Optical	Food quality control, lactose intolerance testing	[5]
Dopamine	Tyrosinase, Aptamers, MIPs	Electrochemical (Voltammetric, Amperometric), Optical	Neurological disorder diagnosis (Parkinson’s, schizophrenia)	[6]
Uric acid	Uricase, Aptamers	Electrochemical (Amperometric, Voltammetric)	Gout and kidney disorder diagnosis	[7]
Cholesterol	Cholesterol oxidase, Cholesterol esterase	Electrochemical (Amperometric, Potentiometric), Optical	Cardiovascular risk assessment	[8]
Lactic acid	Lactate oxidase	Electrochemical (Amperometric), Optical	Sports medicine, sepsis monitoring	[9]
ATP	Aptamers, Enzymes	Luminescent, Electrochemical	Cellular metabolism, cancer detection	[10]
Cortisol (Hormone)	Antibodies, Aptamers, MIPs	Electrochemical (Impedimetric), Optical (SPR, fluorescence)	Stress monitoring, endocrine disorder detection	[11]
Estrogen	Antibodies, Aptamers, MIPs	Electrochemical, Optical	Reproductive health, cancer diagnostics	[12]
Insulin	Antibodies, Aptamers	Electrochemical (Impedimetric), Optical	Diabetes management	[13]
Drugs (e.g., Antibiotics, Narcotics)	Aptamers, Antibodies, Enzymes	Electrochemical (Voltammetric), Optical (SPR, Fluorescence)	Drug abuse detection, therapeutic drug monitoring	[14,15]
Heavy metals (Pb^2+^, Hg^2+^, Cd^2+^)	DNAzymes, Aptamers	Electrochemical, Optical	Environmental monitoring, food/water safety	[16]
Pesticides (e.g., organophosphates)	Acetylcholinesterase (AChE)	Electrochemical (Amperometric), Optical	Agricultural and environmental safety	[17]
Pathogenic bacteria (E. coli, Salmonella)	Antibodies, Aptamers, Bacteriophages	Electrochemical (Impedimetric), Piezoelectric, Optical	Food safety, clinical diagnostics	[18]
Viruses (SARS-CoV-2, Influenza, HIV)	Antibodies, Aptamers, DNA probes	Electrochemical, Optical (SPR, plasmonic), Piezoelectric (QCM)	Infectious disease detection	[19]
DNA (genetic targets)	DNA probes, CRISPR-Cas systems	Electrochemical, Optical (FRET, SPR)	Genetic testing, personalized medicine	[20]
RNA (viral genomes, miRNA)	RNA aptamers, CRISPR-Cas	Electrochemical (Voltammetric), Optical (Fluorescence)	Viral diagnostics, cancer biomarker detection	[21]
CRP (C-reactive protein)	Antibodies, Aptamers	Electrochemical (Impedimetric), Optical (SPR)	Inflammation monitoring, cardiovascular risk	[22]
Troponin (Cardiac biomarker)	Antibodies, Aptamers	Electrochemical (Impedimetric), Optical	Heart attack (myocardial infarction) diagnosis	[23]
Cytokines (IL-6, TNF-α)	Antibodies, Aptamers	Electrochemical, Optical	Immune response monitoring, inflammatory disease	[24]

**Table 3:** List of studies with biosensing applications on electrophysiological signals by 3D-printed biosensors.

S. No.	Biosensing Application	3D Printing Method	Statistical Data/Performance	Advantage	Social/Environmental Impact	Reference
**ELECTROCARDIOGRAM (ECG)**						
1	Self-healable hydrogel–liquid metal ECG sensor	Custom 3D-printed molds	Effective ECG signal acquisition	Self-healing, flexible electrodes	Longer device lifespan, reduced waste	[158]
2	ECG and EEG dry electrode recording	3D-printed electrode arrays	High resolution, repeatable	Affordable, scalable vs. gel electrodes	Accessible monitoring, low-cost healthcare	[159]
3	Multifunctional wearable biosensing (EEG, EOG, motion, and UV)	3D-printed eyeglass frame	Demonstrated integrated biosensing	Customizable, multifunctional	Enhances personal healthcare and HMI	[160]
4	Underwater EEG sensing (zebrafish)	3D-printed multichannel arrays	Feasible in aquatic conditions	Enables biosensing in non-human species	Advances marine neuroscience	[161]
5	On-body biosensing (aerogels)	Freeform closed-loop 3D printing	Functional printing on skin	Prints on moving, curved surfaces	Real-time, non-invasive monitoring	[80]
6	On-tissue electrical impedance sensing	Closed-loop 3D printing on deformable surfaces	Real-time EIT on porcine lung	Compensates for motion, deformation	Improves surgical and diagnostic tools	[162]
7	Smart clothing (ECG/EMG)	Photocuring-based 3D printing of graphene/polymer	Flexible, stretchable electrodes	Wearable, washable integration	Eco-friendly smart textiles	[153]
8	Wearable strain and heartbeat sensors	3D printing of injectable DN hydrogels	Biocompatible, adhesive, tough	Better flexibility and adhesion vs. gels	Safer, reusable wearable healthcare	[163]
9	Remote ECG monitoring (Holter)	3D-printed casing and electronics	Mobile app, SMS, GPRS enabled	Cost-effective, user-friendly	Remote healthcare, reduced hospital visits	[164]
10	Newborn ECG monitoring	3D-printed dry electrodes	92.1% accuracy (rapid HR)	Non-invasive, safe for neonates	Supports remote infant care	[165]
11	Customizable ECG electrodes	FFF with copper-based filament	Flat designs are optimal for conductivity	Adjustable structures for performance	Lower cost, reusable	[166]
12	Veterinary ECG (canines)	3D-printed fur-friendly electrodes	In vivo trials: equivalent to sticky electrodes	Non-invasive, reusable	Enhances animal welfare, reduces waste	[167]
13	Subcutaneous ECG implants (animals)	Custom conductive 3D-printed electrodes	Comparable to commercial implants	Miniaturized, customizable	Biomedical and veterinary research boost	[168]
14	Flexible ECG/EMG biosensors	DLP printing of PEDOT inks	Conductivity: 10^−1^–10^−2^ S/cm	Superior to Ag/AgCl electrodes	Biocompatible, flexible wearables	[169]
**ELECTROENCEPHALOGRAM (EEG)**						
1	EEG monitoring (SSVEPs)	Direct 3D printing of conductive flexible materials	Optimized electrical and mechanical performance	Low-cost, flexible, customizable electrodes	Affordable brain–computer interface applications	[170]
2	EEG and ECG monitoring in small animals	3D-printed biosignal sensor fabrication	Time and cost-efficient fabrication	Alternative to microfabrication, non-invasive	Advances animal studies with lower cost	[141]
3	EMG, EDA, EEG, and strain sensing	High-resolution 3D printing with sugar scaffolds	High sensitivity, precision	Custom-fit, flexible, multimodal sensing	Promotes personalized wearable health tech	[110]
4	Neurocardiology wearable biosensing	3D fabrication of flexible fractal-based sensors	Demonstrated functional wearable system	Low-cost, fractal design improves flexibility	Expands neurocardiology and remote healthcare	[171]
5	EEG monitoring	3D printing of flexible, conformable sensors	Comparable signal quality to commercial electrodes	Enhanced comfort, long-term usability	Improves patient compliance in long studies	[172]
6	EEG monitoring	3D printing with Ag/AgCl-coated electrodes	Reduced noise, improved impedance	Better performance than earlier 3D-printed sensors	Increases reliability for medical use	[140]
7	EEG monitoring (dry electrodes)	Low-cost 3D printing of dry electrodes	Comparable to wet electrodes	Reusable, cost-efficient, non-invasive	Accessible BCI applications, reduced waste	[173]

**Table 4:** List of studies with biosensing applications on biochemical signals by 3D-printed biosensors.

S. No.	Biosensing Application	3D Printing Method	Statistical Data/Performance	Advantage	Social/Environmental Impact	Reference
**GLUCOSE SENSOR**						
1	Electrochemical tattoo glucose sensor	Direct ink writing (DIW)	Sensitivity: 17.5 nA M^−1^; Range: 100–1000 µM	High sensitivity and specificity vs. screen printing	Non-invasive, wearable, and enhances continuous health monitoring	[185]
2	Glucose/lactose ratio in athletes	3D-printed microfluidics (unspecified)	Real-time tissue metabolite tracking	Miniaturization, portability vs. conventional probes	Promotes athlete safety and performance monitoring	[186]
3	Self-powered sweat lactate sensor	Porous carbon film (3D-printed base)	Stable lactate detection with wireless data transfer	Energy autonomy, wearable vs. benchtop assays	Supports sports analytics and big-data-driven health	[187]
4	Multi-analyte biosensor (glucose, lactate, and neurotransmitters)	DIW	Flexible array; compatible with organ-on-chip	Multiplexing vs. single-analyte sensors	Advances neuroscience and clinical diagnostics	[188]
5	In vivo glutamate biosensor	DIW	High signal stability, PtNPs-based electrode	Direct integration, enhanced electrochemical activity	Enables real-time brain monitoring	[189]
6	Neurochemical monitoring (brain)	3D-printed microfluidics	High temporal resolution microdialysis	Portable, integrated vs. bulky lab devices	Supports brain disorder studies and neurology research	[190]
7	Smartphone-enabled glucose biosensor	3D-printed ECL device	Affordable, reagentless glucose detection	Point-of-care adaptability, reagent-free	Improves accessibility in low-resource settings	[191]
8	Photonic glucose sensor	DLP micro-3D printing	Sensitivity: 0.206 nm/mM; linear response	Optical detection vs. enzymatic electrochemistry	Environmentally friendly (UV-cured hydrogel); reusable	[192]
9	Liver-on-a-chip glucose biosensor	FDM with conductive PLA + MWCNT	Enhanced sensitivity via nanocomposites	Low-cost fabrication vs. lithography	Sustainable bioprinting; organ-on-chip integration	[193]
10	GDH-based glucose biosensor	3D printing (unspecified)	Meets industrial performance standards	Robustness, manufacturability vs. manual assembly	Supports scalable diabetic treatment solutions	[194]
11	Disposable non-enzymatic glucose sensor	3D-printed support + MWCNT/NiOOH	Stable electrochemical signals	Enzyme-free, cost-effective vs. enzymatic tests	Disposable design reduces costs and broadens testing access	[195]
**OXYGEN SENSOR**						
1	Finger/toe wearable pulse oximeter	Freeform embedding (FRE) printing with PDMS	PDMS cuff customized to patient anatomy; accurate SpO_2_ and pulse monitoring	Patient-specific fit; better comfort and accuracy than rigid commercial probes	Reduces clinical device waste via custom fabrication; improves patient compliance	[85]
2	Flexible wireless smart bandage for wound oxygenation	3D printing with TangoPlus (FLX930)	Bandage integrates a galvanic oximeter + printed elastomer; continuous wound oxygenation monitoring	Wearable, non-invasive wound care; replaces bulky equipment	Supports remote therapy for chronic wounds, reduces hospital visits	[196]
3	Blood pressure and oxygen monitoring wristband	Direct ink writing (DIW)	Substrate + electrodes printed via DIW; surface mount electronics assembled; integrated platform	Combines biosensing and electronics in one step; lightweight vs. traditional cuffs	Promotes home healthcare and reduces clinical dependency	[197]
4	Photonic biosensor for 3D cell culture (iPOB)	3D-printed chamber with integrated biosensor (unspecified)	Phosphorescence-based oxygen monitoring; 3D-printed culture chamber allows gas exchange	High-resolution, non-invasive cell monitoring; better than manual sampling	Advances biomedical research while minimizing chemical waste	[198]
5	IoT-enabled photometric biosensor system (MAX30102)	3D-printed case with MAX30102 sensor	Continuous SpO_2_ and HR monitoring; integrated with ESP32 + webserver for IoT	Portable, low-cost, real-time remote monitoring vs. hospital devices	Expands access to point-of-care diagnostics; low environmental burden	[199]
**SWEAT SENSOR**						
1	Sweat electrolyte monitoring (multi-ion, real-time)	3D printing of flexible bioelectronic patch (AIIW)	Real-time multi-ion tracking in human sweat	Low-cost, customizable, continuous biochemical monitoring	Noninvasive health tracking; democratizes personalized medicine	[100]
2	Cortisol detection for stress monitoring	3D-printed microfluidic mold + laser-burned graphene with MXene	Continuous cortisol quantification in sweat	High sensitivity, non-invasive stress biosensing	Reduces reliance on blood tests; stress monitoring for mental health	[200]
3	Cytokine detection in serum	Aerosol Jet Printing (AJP) of graphene ink on polyamide	High sensitivity in real samples	Label-free, flexible immunosensing	Enables inflammation monitoring; minimal sample prep	[201]
4	Glucose detection in sweat	3D-printed voltammetric sensor with Fe(III)-cluster	Enzyme-free, stable response under acidic sweat	Cost-effective, avoids enzyme instability	Portable, low-cost diabetes screening	[177]
5	Sweat analyte collection and analysis	Multi-Jet Modeling (MJM) with flexible polymers	Real-time sweat biofluid acquisition	Rapid, direct-on-skin collection	Enhances wearable diagnostics; reusability reduces waste	[202]
6	Sweat sample segmentation and spatial analysis	Digital Light Processing (DLP) for fluidic channels	Multi-compartment sweat capture (“sweatainer”)	Enables parallel analysis of different analytes	Advanced diagnostics, scalable to public health	[203]
7	Multimodal sensing (alcohol inhibition, behavior)	Extrusion-based 3D printing of elastic e-skin (e3-skin)	Continuous multimodal data; ML for behavioral prediction	Integrates biochemical + behavioral sensing	Supports substance abuse monitoring and safety	[204]
8	Smartphone-linked cortisol monitoring	Compact 3D-printed origami microfluidic sensor	Portable, low-cost, real-sweat analysis	Easy integration with smartphones	Expands access to stress diagnostics globally	[205]

**Table 5:** List of studies with biosensing applications on the vascular system by 3D-printed biosensors.

S. No.	Biosensing Application	3D Printing Method	Statistical Data/Performance	Advantage	Social/Environmental Impact	Reference
Blood Pressure Sensor						
1	Ferroelectric artificial artery for BP and occlusion monitoring	Electric field-assisted 3D printing	In situ-poled artery with ferroelectric properties; real-time, battery-free BP sensing; thrombosis detection	Tissue-mimicking modulus; self-powered sensing, unlike battery-dependent cuffs	Reduces device replacement waste; improves patient safety through early clot detection	[213]
2	Wireless pressure sensor in a smart stent	3D-printed biocompatible polymer stent + MEMS	Pressure sensor integrated into a stent, enabling wireless recording of biological signals	Combines structural implant + sensor; avoids invasive monitoring post-surgery	Enables continuous monitoring for cardiac patients; reduces the need for hospital readmission	[214]
3	Wearable ring sensor for BP waveform monitoring	3D printing of ring housing + embedded MEMS	MEMS piezo-resistive sensor in 3D-printed ring; monitors BP waveforms and HRV	Comfortable, long-term use; Better fidelity than cuff-based devices	Promotes at-home monitoring; lowers healthcare system burden	[209]

**Table 6:** List of studies with biosensing applications on signals due to body mechanical deformation (strain sensor), touch sense (tactile sensor), and other miscellaneous physiological signals by 3D-printed biosensors.

S. No.	Biosensing Application	3D Printing Method	Statistical Data/Performance	Advantage	Social/Environmental Impact	Reference
**STRAIN SENSOR**						
1	Human joint motion monitoring	DIW with AGF/CF in PDMS	GF 8–10; FFT for load distinction	High stability, accurate joint tracking	Non-invasive rehab monitoring	[215]
2	Antenna-based strain sensing	FDM with Ninjaflex + ECA	Detects strain via antenna signal loss	Wireless, antenna-integrated sensing	Low-cost and scalable with consumer FDM	[216]
3	Motion and gesture detection	Embedded 3DP (e-3DP)	Reliable under 0–100% strain cycles	Liquid ink encapsulated, robust	Supports prosthetics and human–computer interaction	[78]
4	Human joint motion tracking	3D printing of liquid metal in silicone	>375 cycles at 200% strain; near-zero hysteresis	Highly stretchable and durable	Safer for long-term wearable use	[217]
5	Wearable motion monitoring	Extrusion printing of MWCNT/PDMS	Strain up to 146%; GF = 12.15	High linearity and stretchability	Promotes next-gen fitness/rehab devices	[97]
6	General wearable strain sensing	DIW with nanosilica-modified silicone	Tunable rheology; improved printability	Faster, accurate elastomer fabrication	Optimizes material efficiency	[79]
7	Structural and wearable monitoring	Aerosol Jet Printing (AgNP ink)	Optimized grid design; high precision	High-resolution, miniaturized sensors	Reduced waste via an additive approach	[218]
8	Wearable home healthcare	AJP + laser sintering on a bandage	Stable over 700 bending cycles	Low-cost, disposable, biocompatible	At-home continuous monitoring	[219]
9	Skin motion detection	Inkjet printing PEDOT:PSS + AuNP	GF 0.73 ± 0.1; 0–6% strain; ~1 μm thickness	Ultra-thin, epidermal precision	Minimally invasive, reduced material use	[220]
10	Structural health monitoring	AJP on Buckypaper (CNT)	High conductivity and flexibility	Direct integration in composites	Extends infrastructure lifetime	[221]
11	Motion detection (array)	DLP with UV-curable MWCNT/elastomer	Linear 0.01–45% strain; GF ≈ 8.94	Multi-point, flexible, resilient	Supports robotics and wearable analytics	[222]
12	Robust wearable biomonitoring	FDM sacrificial molds + graphene coating	GF = 10 at 2–10% strain; >75% strain durability	Resistant to solvents and harsh cycles	Sustainable via mold reusability	[223]
13	Human joint motion detection	Material Jetting (silicone + CF)	High GF; flexible and foldable	Precise drop-on-demand fabrication	Energy efficient, scalable	[224]
14	Strain + VOC gas sensing	DIW TPU/CB foam	Linear up to ~80% strain; selective VOC response	Dual sensing capability (strain + gas)	Environmental VOC detection + wearable use	[103]
15	High-precision monitoring	DIW graphene/PDMS composite	Stable GF after 100 cycles	High sensitivity and repeatability	Enables precision diagnostics	[225]
16	Selective stretch/bend sensing	3D elastomer molds + agarose ionic gel	GF = 17; up to 500% strain	Biocompatible, high stretch selectivity	Eco-friendly ionic materials	[226]
17	Strain and pressure sensing	DLP hydrogel (PAAm-PEGDA)	High sensitivity; static and dynamic detection	Capacitive, flexible, multi-sensing	Sustainable hydrogels for wearables	[227]
**TACTILE SENSOR**						
1	Capacitive touch sensing on curved 3D surfaces	Aerosol jet printing (AJP) of AgNPs ink	Functional sensors on ABS, PC, PVC	Integrates on complex geometries	Expands IoT and robotics interfaces	[228]
2	Finger motion and pulse monitoring	Customized 3D printing on freeform surfaces	Skin-conforming detection of motion/pulse	Flexible, wearable integration	Enhances personalized health tracking	[229]
3	Soft pressure sensing (acoustic/pulse)	Inkjet printing of AgNPs on PDMS	Sensitivity: 0.48 kPa^−1^	High reproducibility, wearable	Improves low-cost health electronics	[230]
4	Ultrathin vibration sensing	Direct ink writing (DIW) + chemical reduction	Detects subtle vibrations and weak pulses	Stretchable, ultra-thin electrodes	Wearable for biomedical and robotics	[231]
5	Strain and humidity sensing	Aerosol jet printing (Pt/AgNP inks, free-standing films)	Highly flexible free-standing structures	Enables multifunctional sensing	Supports sustainable wearable systems	[232]
6	Wearable tactile sensing (high strain tolerance)	DIW with PDMS/GO nanocomposite	Strain range ~40%, low resistivity	Improved mechanical robustness	Durable and reduces sensor replacement	[233]
7	Piezo-resistive tactile sensing	FDM with conductive filament	Achieved SINAD = 18 dB	Low-cost, 3D-printed, flexible	Scalable for robotics and prosthetics	[234]
8	Ionic pressure sensing (ultra-low pressure, pulse)	3D-printed ordered hierarchical mesh	Sensitivity: 72.86 kPa; Durability: 7300 cycles	Tunable and durable	Real-time health + communication tools	[235]
9	Dual-mode resistive/capacitive pressure sensing	Extrusion printing of CNT-elastomer	Capacitive: 0.02 kPa, 25 ms; Resistive: 5 Pa, 20 ms	Rapid, multimodal detection	Useful for prosthetics and HMI devices	[236]
10	Integrated pressure and strain sensing	Coaxial extrusion AM of fibers	Detects shear, twist, bend, and press	Multifunctional e-skin	Human–machine interaction, robotics	[237]
11	Health monitoring, tissue engineering	Single-component CNT–silicone ink	High conductivity and flexibility	Simplifies fabrication	Reusable, supports medical bioplatforms	[196]
12	Tactile sensing + energy harvesting	Inkjet + DIW of triboelectric nanogenerator	All-printed TENG, tactile + power gen	Energy self-sufficient	Reduces battery waste in wearables	[238]
13	Multi-parameter sensing (force, temp, gas)	Mold-based 3D printing with PDMS/graphite	Low-force sensing patches	Low-cost, multi-signal monitoring	Affordable environmental diagnostics	[239]
14	Self-powered tactile sensing	3D printing of soft triboelectric materials	Distinct responses to force/frequency	Operates without batteries	Promotes sustainable e-skin devices	[240]
15	Microforce sensing (µN resolution)	FDM + SLA	Detects micro-Newton forces	High sensitivity, customizable	Useful for biomedical microsurgery	[241]
16	Real-time wearable monitoring (respiration, pulse)	All-3D-printed hybrid nanocomposite sensors	Monitors multiple signals	Low-cost, biocompatible	Expands access to wearable healthcare	[242]
17	Breast cancer identification	3D-printed tactile probe + FBG sensors	Improved force sensitivity, non-invasive	Overcomes the limits of manual palpation	Early cancer screening reduces biopsies	[243]
**MISCELLANEOUS**						
1	RF electronics and sensors for biomonitoring	Inkjet/3D/4D printing on paper and polymer substrates	Demonstrated scalable RF modules	Low-cost, flexible, system-level integration	Enables affordable, wide-access wearable biomonitoring	[244]
2	Wearable smart health and food quality sensors	SLA 3D printing + metallization	Good sensitivity, IoT-enabled	Combines SIW and microfluidics, flexible design	Supports IoT in healthcare and food safety	[245]
3	Oxidative stress monitoring (protein carbonylation)	3D printing of optical fiber biosensors	Dynamic in vivo protein carbonyl detection	Real-time, non-invasive stress monitoring	Applications in chronic disease, sports, and livestock health	[246]
4	Wearable biomedical devices and electronic tattoos	Aerosol jet printing (AJP) of silver nanowires	High conductivity, strong adhesion	Ultra-thin, flexible, fast drying	Eco-friendly, reusable e-tattoos for health monitoring	[247]

## Data Availability

The data presented in this study are available on request from the corresponding author.

## Supplementary Materials

The following supporting information can be downloaded at: https://www.mdpi.com/article/10.3390/bios15090619/s1, Table S1. Classification of biosensors: components and detection method.
