# Supplementary material for: 3D Printing Assisted Wearable and Implantable Biosensors
Source: Biosensors (Basel). 2025 Sep 17;15(9):619. doi: 10.3390/bios15090619 (PMC12468503; doi:10.3390/bios15090619)
Supplement: Supplementary file 1 [file biosensors-15-00619-s001.zip › biosensors-3786636-supplementary.pdf]

3D Printing Assisted Wearable and Implantable Biosensors

Somnath Maji [1,†], Myounggyu Kwak [2,†], Reetesh Kumar [3], Hyungseok Lee [2,4,*]

[1] Department of Radiology, University of Michigan, Ann Arbor 48109, MI, USA; smaji@umich.edu
[2] Department of Smart Health Science and Technology, Kangwon National University (KNU), Chuncheon-si 24341, Republic of Korea; eric7947@kangwon.ac.kr
[3] Department of Bioengineering and Biotechnology, School of Basic and Applied Sciences, Galgotias University, Greater Noida 203201, India; reetesh.kumar@galgotiasuniversity.edu.in
[4] Department of Mechanical and Biomedical Engineering, Kangwon National University (KNU), Chuncheon-si 24341, Republic of Korea.
* Correspondence: ahl@kangwon.ac.kr
† Authors with equal contribution

Table S1. Classification of Biosensors: Components and Detection Methods.

Component – Bioreceptor				
**Bioreceptor Type**	**Description**	**Advantages**	**Limitations**	**References**
Enzymes	Biological catalysts with redox-active sites suitable for direct electron transfer	High specificity, catalytic activity enhances sensitivity, cost-efficient	Limited stability, affected by environmental conditions	[1,2]
Antibodies	Proteins that bind specifically to target antigens	Excellent specificity for complex molecules, well-established immobilization techniques	Production complexity, potential cross-reactivity	[3]
Nucleic Acids	DNA or RNA sequences that recognize complementary strands	High specificity through hybridization, capable of detecting genetic mutations	Requires sample preparation, sensitivity to degradation	[4]
Whole Cells	Intact microorganisms that respond to environmental changes	Detect complex biological effects, simulate in vivo responses	Maintenance challenges, lower specificity	[5]
**Component – Transducer**				
Transducer Type	***Signal Conversion***	***Characteristics***	***Applications***	***References***
Electrochemical	Biological reactions to electrical signals	High sensitivity, miniaturization potential, direct electrical interface	Disease biomarkers, environmental pollutants	[2,3]
Optical	Biological interactions to light property changes	Label-free detection, real-time monitoring capabilities	Protein-protein interactions, clinical diagnostics	[4,6]
Piezoelectric	Mass changes to frequency shifts	High precision for mass detection, suitable for label-free assays	Virus detection, protein binding studies	[7]

Nanomaterial-Based	Enhanced signal transduction through nanoscale properties	Improved sensitivity, larger active surface area	Multiple biosensing applications	[8]
**Detection Method - Electrochemical**				
Method	***Principle***	***Sensitivity***	***Target Analytes***	***References***
Direct Electron Transfer (DET)	Mediator-free electron exchange between redox enzymes and electrodes	High specificity without interference from mediators	Glucose, lactose, other small molecules	[1]
Amperometric	Measures current produced by redox reactions	High sensitivity at low concentrations	Glucose, food toxins, heavy metals	[3,9]
Potentiometric	Measures potential difference at zero current	Good for ionic species detection	pH changes, ion concentrations	[10]
Impedimetric	Measures impedance changes	Effective for surface binding events	Antibody-antigen interactions	[11]
**Detection Method - Optical**				
Method	***Principle***	***Characteristics***	***Applications***	***References***
Surface Plasmon Resonance (SPR)	Light-induced electron oscillations at metal-dielectric interfaces	Label-free, real-time kinetic measurements	Biomolecular interactions, affinity studies	[12]
Localized SPR (LSPR)	Confined plasmon oscillations in metal nanostructures	Enhanced sensitivity, smaller sensing volumes	Protein detection, biomarker analysis	[13]
Long-Range SPR (LRSPR)	Extended propagation of surface plasmons	Improved sensitivity and detection range	Trace analyte detection	[14]
Fiber Optic-Based	Light propagation through optical fibers	Remote sensing capability, immunity to electromagnetic interference	Environmental monitoring, biochemical sensing	[15]

Resonant Mirror/Waveguide	Light coupling into resonant structures	High precision for thin film analysis	Protein binding studies	[16]
**Other Detection Methods**				
Method	***Principle***	***Key Features***	***Applications***	***References***
Piezoelectric/Acoustic	Mass-sensitive frequency changes	High sensitivity to surface mass changes	Virus detection, pathogen identification	[17]
Magnetostrictive	Magnetic field-induced mechanical changes	Remote actuation and sensing	Virus detection in complex matrices	[7]
Dual Polarization Interferometry	Phase shift in polarized light	High precision for structural analysis	Protein conformational changes	[18]
Machine Learning Enhanced	Algorithmic pattern recognition from sensor data	Improved classification accuracy, handling of complex datasets	Cell classification, multiparameter analysis	[19]

References

1. Ahmad, F.; Zahid, S.; Khan, M.I.; Shanableh, A.; Farooq, N.; Rao, K.A.; Taj, M.B.; Manzoor, S.; Voskressensky, L.G.; Luque, R. Direct Electron Transfer Chemistry of Redox-active Enzymes: Applications in Biosensor Development. *Biofuels Bioprod Bioref* **2025**, *19*, 963–981, doi:10.1002/bbb.2742.
2. A Review of Biosensors and Their Applications. *ASME Open Journal of Engineering* **2023**, *2*, 020201, doi:10.1115/1.4063500.
3. Feng, J.; Chu, C.; Ma, Z. Electrochemical Signal Substance for Multiplexed Immunosensing Interface Construction: A Mini Review. *Molecules* **2022**, *27*, 267, doi:10.3390/molecules27010267.
4. Daghestani, H.N.; Day, B.W. Theory and Applications of Surface Plasmon Resonance, Resonant Mirror, Resonant Waveguide Grating, and Dual Polarization Interferometry Biosensors. *Sensors* **2010**, *10*, 9630–9646, doi:10.3390/s101109630.
5. Aschenbrenner, G.; Goswami, S.; Usmani, K.; Javidi, B. Review: Advances in Lensless Random Phase Encoded Imaging for Automated Cell Identification. *Opt. Eng.* **2024**, *63*, doi:10.1117/1.OE.63.11.111814.
6. Wang, Q.; Zhang, D.; Qian, Y.; Yin, X.; Wang, L.; Zhang, S.; Wang, Y. Research on Fiber Optic Surface Plasmon Resonance Biosensors: A Review. *Photonic Sens* **2024**, *14*, doi:10.1007/s13320-024-0703-7.
7. Narita, F.; Wang, Z.; Kurita, H.; Li, Z.; Shi, Y.; Jia, Y.; Soutis, C. A Review of Piezoelectric and Magnetostrictive Biosensor Materials for Detection of COVID-19 and Other Viruses. *Advanced Materials* **2021**, *33*, doi:10.1002/adma.202005448.
8. Wei, Y. Advances on the Biosensor Based on Nanotechnology. *J. Phys.: Conf. Ser.* **2021**, *1885*, 022023, doi:10.1088/1742-6596/1885/2/022023.
9. Hayat, A.; Catanante, G.; Marty, J. Current Trends in Nanomaterial-Based Amperometric Biosensors. *Sensors* **2014**, *14*, 23439–23461, doi:10.3390/s141223439.

10. Karimi-Maleh, H.; Orooji, Y.; Karimi, F.; Alizadeh, M.; Baghayeri, M.; Rouhi, J.; Tajik, S.; Beitollahi, H.; Agarwal, S.; Gupta, V.K.; et al. A Critical Review on the Use of Potentiometric Based Biosensors for Biomarkers Detection. *Biosensors and Bioelectronics* **2021**, *184*, 113252, doi:10.1016/j.bios.2021.113252.
11. Štukovnik, Z.; Bren, U. Recent Developments in Electrochemical-Impedimetric Biosensors for Virus Detection. *IJMS* **2022**, *23*, 15922, doi:10.3390/ijms232415922.
12. Wang, Q.; Ren, Z.-H.; Zhao, W.-M.; Wang, L.; Yan, X.; Zhu, A.; Qiu, F.; Zhang, K.-K. Research Advances on Surface Plasmon Resonance Biosensors. *Nanoscale* **2022**, *14*, 564–591, doi:10.1039/d1nr05400g.
13. Unser, S.; Bruzas, I.; He, J.; Sagle, L. Localized Surface Plasmon Resonance Biosensing: Current Challenges and Approaches. *Sensors* **2015**, *15*, 15684–15716, doi:10.3390/s150715684.
14. Jing, J.; Liu, K.; Jiang, J.; Xu, T.; Wang, S.; Ma, J.; Zhang, Z.; Zhang, W.; Liu, T. Double-Antibody Sandwich Immunoassay and Plasmonic Coupling Synergistically Improved Long-Range SPR Biosensor with Low Detection Limit. *Nanomaterials* **2021**, *11*, 2137, doi:10.3390/nano11082137.
15. Wang, X.; Wolfbeis, O.S. Fiber-Optic Chemical Sensors and Biosensors (2015–2019). *Anal. Chem.* **2020**, *92*, 397–430, doi:10.1021/acs.analchem.9b04708.
16. Kuo, C.-W.; Wang, S.-H.; Lo, S.-C.; Yong, W.-H.; Ho, Y.-L.; Delaunay, J.-J.; Tsai, W.-S.; Wei, P.-K. Sensitive Oligonucleotide Detection Using Resonant Coupling between Fano Resonance and Image Dipoles of Gold Nanoparticles. *ACS Appl. Mater. Interfaces* **2022**, *14*, 14012–14024, doi:10.1021/acsami.1c21936.
17. Mészáros, G.; Akbarzadeh, S.; De La Franier, B.; Keresztes, Z.; Thompson, M. Advances in Electromagnetic Piezoelectric Acoustic Sensor Technology for Biosensor-Based Detection. *Chemosensors* **2021**, *9*, 58, doi:10.3390/chemosensors9030058.
18. Escorihuela, J.; González-Martínez, M.Á.; López-Paz, J.L.; Puchades, R.; Maquieira, Á.; Gimenez-Romero, D. Dual-Polarization Interferometry: A Novel Technique To Light up the Nanomolecular World. *Chem. Rev.* **2015**, *115*, 265–294, doi:10.1021/cr5002063.
19. Raji, H.; Tayyab, M.; Sui, J.; Mahmoodi, S.R.; Javanmard, M. Biosensors and Machine Learning for Enhanced Detection, Stratification, and Classification of Cells: A Review. *Biomed Microdevices* **2022**, *24*, doi:10.1007/s10544-022-00627-x. 19
